# Synchronized activity of sensory neurons initiates cortical synchrony in a model of neuropathic pain

**DOI:** 10.1038/s41467-023-36093-z

**Published:** 2023-02-08

**Authors:** Chao Chen, Linlin Sun, Avital Adler, Hang Zhou, Licheng Zhang, Lihai Zhang, Junhao Deng, Yang Bai, Jinhui Zhang, Guang Yang, Wen-Biao Gan, Peifu Tang

**Affiliations:** 1 Department of Orthopaedics, Peking 301 Hospital, Beijing, China; 2 Department of Hand Surgery, Shenzhen People’s Hospital, Second Clinical Medicine College of Jinan University, First Affiliated Hospital of Southern University of Science and Technology, Shenzhen, Guangdong China; 3 Department of Anesthesiology, Columbia University Medical Center, New York, NY USA; 4 Department of Neurobiology, School of Basic Medical Sciences, Key Laboratory for Neuroscience, Ministry of Education/National Health Commission of China, Neuroscience Research Institute, Peking University, Beijing, China; 5 Skirball Institute, Department of Neuroscience and Physiology, New York University School of Medicine, New York, NY USA; 6 Institute of Neurological and Psychiatric Disorders, Shenzhen Bay Laboratory, Shenzhen, Guangdong China; 7 Department of Orthopaedics, the Affiliated Southeast Hospital of Xiamen University, Zhangzhou 175 Hospital, Zhangzhou, Fujian China

**Keywords:** Chronic pain, Somatic system, Sensory processing

## Abstract

Increased low frequency cortical oscillations are observed in people with neuropathic pain, but the cause of such elevated cortical oscillations and their impact on pain development remain unclear. By imaging neuronal activity in a spared nerve injury (SNI) mouse model of neuropathic pain, we show that neurons in dorsal root ganglia (DRG) and somatosensory cortex (S1) exhibit synchronized activity after peripheral nerve injury. Notably, synchronized activity of DRG neurons occurs within hours after injury and 1-2 days before increased cortical oscillations. This DRG synchrony is initiated by axotomized neurons and mediated by local purinergic signaling at the site of nerve injury. We further show that synchronized DRG activity after SNI is responsible for increasing low frequency cortical oscillations and synaptic remodeling in S1, as well as for inducing animals’ pain-like behaviors. In naive mice, enhancing the synchrony, not the level, of DRG neuronal activity causes synaptic changes in S1 and pain-like behaviors similar to SNI mice. Taken together, these results reveal the critical role of synchronized DRG neuronal activity in increasing cortical plasticity and oscillations in a neuropathic pain model. These findings also suggest the potential importance of detection and suppression of elevated cortical oscillations in neuropathic pain states.

## Abstract (web summary)

Increased low frequency cortical oscillations are observed in neuropathic pain. Here the authors perform Ca^2+^ imaging of DRG sensory neurons in a mouse model of neuropathic pain, and show that synchronized activity of DRG neurons occurs within hours after injury and 1-2 days before increased cortical oscillations are observed.

## Introduction

Neuropathic pain arises as a debilitating consequence of peripheral nerve injury or insult. The mechanisms underlying neuropathic pain remain unclear, and existing treatments are largely ineffective^1^. Low-frequency cortical oscillations are often enhanced in neuropathic pain patients and animal models^2–10^. Activity synchrony of spinal dorsal horn neurons is also augmented under pain condition and after noxious stimuli in rodents^11–14^. In addition, synchronized neuronal activity is elevated between pain-related spinal^15^ and supraspinal regions^7,16–18^. In the primary somatosensory cortex (S1), layer 2/3 (L2/3) pyramidal neurons undergo a prolonged period of increased basal activity during sustained pain states^19,20^ that is associated with an increase in synchronization^21,22^. Although the increase of neuronal synchronization is widespread during the development of neuropathic pain, the mechanisms and functional impact of such elevated activity synchrony remain unknown.

The primary sensory neurons in dorsal root ganglia (DRG) detect external stimuli and relay the signal to the central nervous system (CNS)^23^. DRG neurons exhibit spontaneous hyperactivity upon nerve injury^24^ or insult^25^, likely due to aberrant signaling at the injured site^26^, as well as maladaptive changes of receptors, enzymes and ion channels in the ganglia^27^. The enhanced DRG neuronal activity could further trigger plastic changes in the upstream pain pathway, including spinal cord, subcortical and cortical regions^27–31^, leading to the development of chronic pain^20,28,31–33^. Nevertheless, whether and how DRG neurons contribute to increased cortical oscillations and plasticity under chronic pain conditions remain unclear.

In this study, we took advantage of a newly-developed method to monitor activity of DRG neurons in awake mice during neuropathic pain development. We show synchronized activation of DRG neurons occurs rapidly in a spared nerve injury (SNI) mouse model of pain. Importantly, the synchrony, not the level, of DRG neuronal activity after SNI is critical for increased cortical plasticity, enhanced cortical oscillations, and pain hypersensitivity. Our findings reveal the important role of DRG activity synchrony in promoting cortical plasticity and oscillations in neuropathic pain development. They also suggest that early detection and suppression of increased cortical oscillations could serve as an important means for the diagnosis and treatment of chronic pain.

## Results

### Peripheral nerve injury increases cortical oscillation and synchronized neuronal activity

**Fig. 1:**
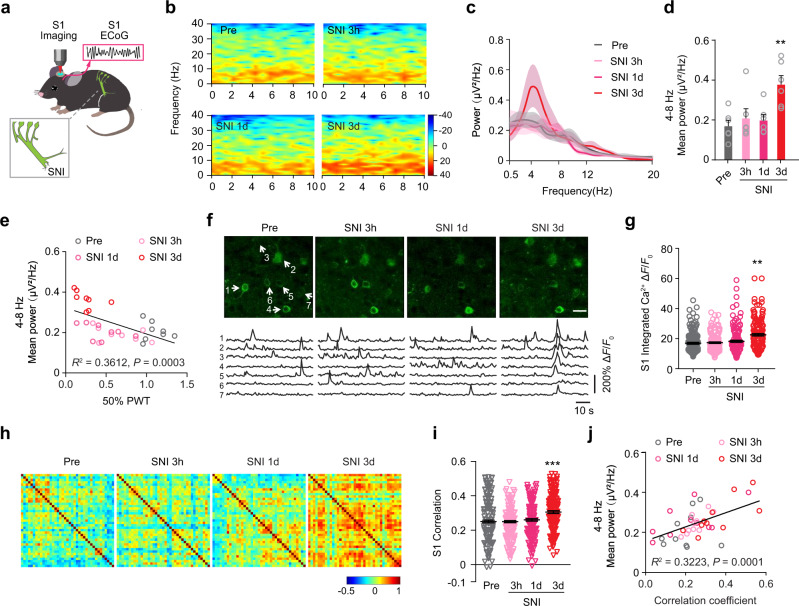
Peripheral nerve injury induces activity synchrony in S1. **a** Schematic showing ECoG recording and Ca^2+^ imaging of L2/3 pyramidal neurons in S1 HL region in awake behaving SNI mice. Pink inset: ECoG recording; gray inset: SNI surgery. **b** Spectrograms of ECoG signals in S1 of awake behaving SNI mice. **c** ECoG power spectra before (Pre), 3 hours, 1 day, and 3 days after SNI. **d** The mean power in theta band (4–8 Hz). **e** ECoG power in theta band correlates inversely with mechanical paw withdraw threshold. **f** Representative two-photon images and fluorescence traces of L2/3 pyramidal neurons in S1 before and after SNI. Arrows point to cells with Ca^2+^ traces shown below. Scale, 50 μm. **g** Integrated Ca^2+^ activity of L2/3 pyramidal neurons in S1. **h** Representative correlation coefficient matrix of calcium transients from all active neuron pairs in L2/3 of S1. **i** Correlation coefficient of L2/3 pyramidal neurons in S1 before and after SNI. **j** Correlation coefficient of L2/3 pyramidal neurons in S1 correlates positively with the mean power in theta band. Data are expressed as mean  ±  SEM. **P < 0.01, *** *P* < 0.001. See Table S1 for statistical details. Source data are provided as a Source Data file.

To explore the origin and function of pain-related low-frequency cortical oscillations, we used a SNI mouse model of neuropathic pain^20,34,35^, in which axotomy of tibial and common peritoneal branches of sciatic nerve resulted in mechanical allodynia (Supplementary Fig. 1a) and ongoing pain behaviors (Supplementary Fig. 1b). By performing electrocorticography (ECoG) recording in the mouse primary somatosensory cortex (S1), we found marked increases in the power of theta (4–8 Hz) band within 3 days, but not 3–24 hours, after SNI (Fig. 1a–d). No significant difference was observed in delta (0.5–4 Hz) and alpha (8–12 Hz) band power within 3 days after SNI (Fig. 1c, Supplementary Fig. 1c). The animals’ paw withdrawal threshold indicative of mechanical allodynia showed a negative correlation with theta band power (Fig. 1e), but no correlation with alpha band power (Supplementary Fig. 1d). Thus, similar to neuropathic pain conditions in patients^4,5,9,10^ and animal models^2,6,7^, increased low-frequency cortical oscillations are associated with pain development in SNI mice.

To better understand enhanced cortical oscillations after SNI, we first examined the activity of layer 2/3 (L2/3) pyramidal neurons in S1 of head-fixed mice with in vivo two-photon Ca^2+^ imaging (Fig. 1a; see methods). We found that the integrated Ca^2+^ activity of L2/3 pyramidal neurons in S1 was significantly higher 3–21 days, but not 3–24 hours, after SNI (Fig. 1f, g, Supplementary Fig. 1e). Using Pearson’s correlation coefficient (R) between the activity of neuronal pairs to evaluate the level of synchronized activity, we found that synchrony of L2/3 pyramidal neuronal activity increased significantly 3–21 days, but not 3–24 hours, after SNI (Fig. 1h, i, Supplementary Fig. 1f). The level of activity synchrony was positively correlated with theta band power (Fig. 1j), but not with alpha band power (Supplementary Fig.1i). In addition, we also observed the increased level and synchrony of L5 pyramidal neuronal activity 3–21 days after SNI (Supplementary Fig.1g, h). Given the crucial role of pyramidal neurons in the generation of EEG/ECoG signals^36,37^, these results suggest that the SNI-induced increase of cortical oscillations in theta band is at least partially due to increased activity synchrony in pyramidal neurons in S1.

### Injury induces synchronized activity in DRG before enhanced cortical oscillation

**Fig. 2:**
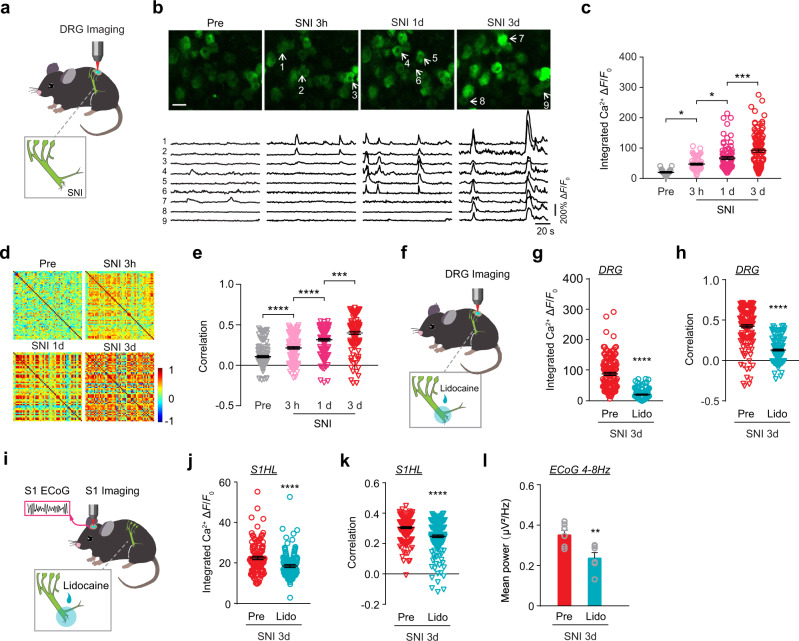
Peripheral nerve injury induces activity synchrony in DRG neurons prior to pyramidal neurons in S1. **a** Schematic showing Ca^2+^ imaging of sensory neurons in L4 DRG in awake mice. Gray inset: SNI surgery. **b** Representative two-photon images and fluorescence traces of L4 DRG sensory neurons before, 3 hours, 1 day, and 3 days after SNI. Arrows point to neurons with Ca^2+^ traces shown below. Scale, 50 μm. **c** The integrated Ca^2+^ activity of active neurons in the DRG before, 3 hours, 1 day, and 3 days after SNI surgery. **d** Representative correlation coefficient matrix of calcium transients from all active neuron pairs in DRG before and after SNI. **e** The correlation coefficient of DRG neurons before and after SNI surgery. **f** Schematic showing Ca^2+^ imaging of sensory neurons in L4 DRG in awake mice treated with peripheral lidocaine. **g**, **h** The integrated Ca^2+^ activity **g** and correlation coefficient (**h**) of DRG neurons pre and post-peripheral lidocaine application in mice 3 days after SNI. **i** Schematic showing ECoG recording and Ca^2+^ imaging of L2/3 pyramidal neurons in S1 in awake mice treated with peripheral lidocaine. **j**–**l** The integrated Ca^2+^ activity (**j**), correlation coefficient of L2/3 pyramidal neurons in S1 (**k**), and mean ECoG power in theta band (**l**) pre and post peripheral lidocaine application in mice 3 days after SNI. Data are expressed as mean  ±  SEM. *P < 0.05, **P < 0.01, ***P < 0.001, ****P < 0.0001. See Table S1 for statistical details. Source data are provided as a Source Data file.

Following nerve injury or inflammation, the excitability of peripheral and cortical neurons in the pain pathway increases over time^20,38–41^. DRG neurons are the first-order sensory neurons for detecting external stimuli and for conveying signals to CNS^42,43^. Suppressing DRG neuronal activity has been shown to ameliorate chronic pain development^44–46^. To investigate the potential contribution of DRG neuronal activity to enhanced cortical activity synchrony, we performed Ca^2+^ imaging of lumbar 4 (L4) DRG sensory neurons in awake mice using a recently developed imaging technique^25^. Imaging was performed after exposing the left L4 DRG and fixing the spine between L3–L5 with a custom-made metal sheet (see methods). We found that the integrated Ca^2+^ activity of DRG neurons was significantly higher within 3 hours after SNI when compared to pre-SNI (Fig. 2a–c; Movie S1). Notably, when compared to pre-SNI, Pearson’s correlation coefficient of DRG neuronal activity was about 2, 3, and 4 times higher at 3 hours, 1 day, and 3 days after SNI (Fig. 2d, e; Supplementary Fig. 2d). These changes were observed in mice after SNI, but not after sham surgery (Supplementary Fig. 2a–f). In addition, the percentage of DRG neuronal pairs displaying significant correlation of activity (see Data Analysis) was substantially higher 1 day and 3 days after SNI as compared to that in sham mice (Supplementary Fig. 2g). The increased level and synchrony of DRG neuronal activity persist over 3 weeks after SNI (Supplementary Fig. 2h, i). Together, these results indicate that the level and synchrony of DRG neuronal activity exhibit rapid (within hours) and persistent increase after SNI, 1–2 days earlier than increased cortical oscillation in S1.

When the sodium and potassium channel blocker lidocaine (1%) was locally applied to the injured sciatic nerve 3 days after SNI, the level and synchrony of DRG neuronal activity reduced significantly (Fig. 2f–h). Concomitantly, the level and synchrony of S1 cortical neuronal activity, as well as ECoG theta band power were suppressed (Fig. 2i–l, Supplementary Fig. 2j). Similar effects were observed after peripheral application of the sodium channel blocker tetrodotoxin (500 nM) to the injured sciatic nerve (Supplementary Fig. 2k–p). In addition, we administered lidocaine to the sciatic nerve three times a day over 3 days after SNI and performed Ca^2+^ imaging in S1 on days 3 and 7 post-SNI (Supplementary Fig. 2q). We found that repeated peripheral lidocaine administration in SNI mice decreased Ca^2+^ activity level and synchrony in L2/3 pyramidal neurons when compared to the controls with saline treatment (Supplementary Fig. 2r, s). Together, these findings strongly suggest that hyper- and synchronized activity in DRG sensory neurons after SNI are important for increased cortical neuronal activity and synchrony, as well as low-frequency oscillations in S1.

### Axotomized neurons initiate DRG activity synchrony

**Fig. 3:**
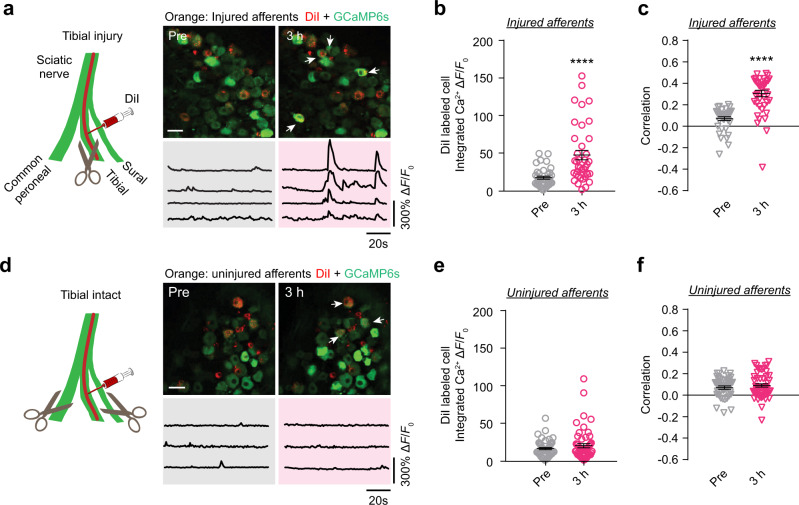
Axotomized neurons are initiators in DRG synchrony. **a** Schematic shows that retrograde nerve tracer DiI was injected into the tibial branch of the sciatic nerve, and tibial nerve was cut and ligated 7 days after injection. Upper right: representative images of double labeled tibial nerve-injured-neurons and GCaMP6 neurons before and 3 hours after tibial nerve cut. Scale, 50 μm. Lower right: fluorescence traces of double labeled tibial nerve-injured-neurons shown in upper right panels. **b**, **c** The integrated Ca^2+^ activity **b** and correlation coefficient **c** of double-labeled neurons before and 3 hours after tibial nerve injury. **d** Schematic shows that DiI was injected into the tibial branch of the sciatic nerve and the other two branches (common peroneal and sural nerves) were cut and ligated 7 days after injection. Upper right: representative images of double labeled tibial nerve-intact neurons before and 3 hours after cutting other two nerves. Scale, 50 μm. Lower right: fluorescence traces of double labeled tibial nerve-intact-neurons shown in upper right panels. **e**, **f** The integrated Ca^2+^ activity (**e**) and correlation coefficient (**f**) of double-labeled neurons before and 3 hours after cutting common peroneal and sural nerves. Data are expressed as mean  ±  SEM. ****P < 0.0001. See Table S1 for statistical details. Source data are provided as a Source Data file.

Given the potential importance of DRG activity in cortical changes after SNI, we further investigated factors contributing to the generation of DRG synchrony after SNI. We first examined if injured peripheral afferents are responsible for eliciting activity changes in DRG. In this experiment, we injected retrograde nerve tracer DiI (red, 0.5% in anhydrous alcohol) into the tibial branch of sciatic nerve in *Thy1*-GCaMP6 mice to retrogradely label their somas in lumbar 4 DRG (Fig. 3a–d). The activity of DiI-labeled GCaMP6-expressing tibial neurons was measured before and after either tibial nerve injury (Fig. 3a–c) or spared tibial nerve injury (Fig. 3d–f). Following tibial nerve transection, the activity level and synchrony of DiI-labeled tibial neurons (injured afferents) increased significantly within 3 hours as compared to preinjury baseline (Fig. 3b, c). In contrast, 3 hours after spared tibial nerve injury (common peroneal and sural branches transected, tibial branch intact) (Fig. 3d), both the activity level and synchrony of DiI-labeled tibial neurons (uninjured afferents) were comparable to their pre-injury baseline (Fig. 3e, f). Thus, axotomized rather than intact neurons exhibit activity changes within hours after peripheral nerve injury and are likely involved in the initiation of DRG activity synchrony. Moreover, correlation coefficient R of axotomized neurons showed a progressive increase within 3 days after SNI, indicating that axotomized neurons contribute to DRG synchrony development (Supplementary Fig 3a).

### ATP released at the traumatic zone initiates DRG synchrony

**Fig. 4:**
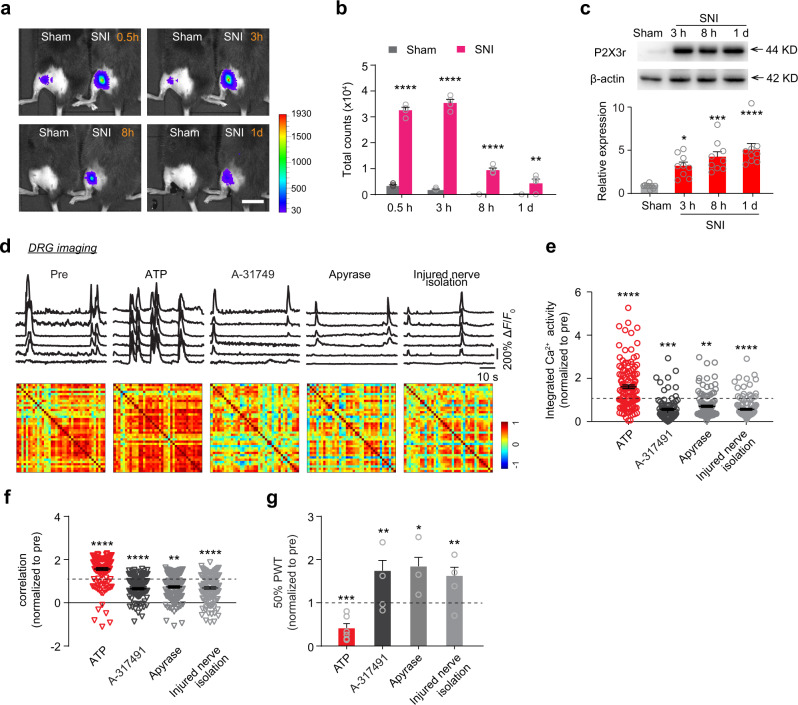
ATP at the site of injury is important for synchronized DRG neuronal activity after SNI. **a** Representative images of ATP release detected by bioluminescence 0.5, 3, 8, and 24 hours after sham or SNI surgery. Scale, 20 mm. The color bar is luminescence signal in counts. **b** The level of luminescence signal from the injured zone at various time points after sham or SNI surgery. **c** Western blot analysis of the expression of P2X_3_ receptor in the injured nerves from SNI mice. **d**–**f** Representative fluorescence traces and correlation matrices (**d**) as well as normalized integrated Ca^2+^ activity (**e**) and correlation coefficient (**f**) of DRG neurons before and after local application of ATP, A-317491, and ATP hydrolyzes (apyrase) to the sciatic nerve, and isolation of injured nerve end in SNI 3d mice. **g** Measurement of hindlimb PWT before and after local application of ATP, A-317491, or apyrase to the sciatic nerve, and injured nerve isolation in SNI 3d mice. Data are expressed as mean  ±  SEM. *P < 0.05, **P < 0.01, ***P < 0.001, ****P < 0.0001. See Table S1 for statistical details. Source data are provided as a Source Data file.

Previous studies have shown that ATP is locally released in the traumatic zone after acute nerve injury and involved in nociceptive transmission^26^. To explore whether ATP at the site of nerve injury may contribute to the elevated level and synchrony of DRG neuronal activity after SNI, we measured the ATP level in living mice by monitoring luminescence resulting from ATP-triggered luciferase breakdown of luciferin^47,48^. Real-time imaging showed that the ATP level after surgery was substantially higher at the injury site in SNI mice as compared to that in sham mice (Fig. 4a, b). In sham mice, low levels of luminescence were observed around skin incision site 0.5 and 3 hours, but not >8 hours after surgery. In SNI mice, the level of luminescence at the injury site increased sharply within 0.5 to 3 hours after SNI and reduced gradually afterwards (Fig. 4a, b), indicating that the high level of ATP release persists for hours following nerve injury.

Injury-induced ATP release could activate axonal purinergic receptors, leading to the excitation of DRG neurons and transduction of nociceptive stimuli^49^. Consistently, western blotting experiments showed that P2X_3_ receptors were highly expressed in peripheral afferents in both sham and SNI groups. Furthermore, a significant up-regulation of P2X_3_ receptor expression in injured fibers was observed 8 and 24 hours after SNI surgery when compared to the sham group (Fig. 4c).

To test whether elevated ATP and P2X_3_ receptors at the site of injury are important for DRG neuronal hyperactivity and synchrony after SNI, we first examined neuronal Ca^2+^ response in DRG following local administration of exogenous ATP (10 mM) to the injured sciatic nerve. Three to five minutes after ATP application in SNI mice, we found a marked increase in the level and synchrony of DRG neuronal activity (Fig. 4d–f). ATP application also exacerbated mechanical allodynia as measured by animals’ paw withdrawal threshold (Fig. 4g). In addition, local application of A-317491 (200 μM), a selective antagonist of P2X_3_ and P2X_2/3_ receptors, to the injured sciatic nerve after SNI reduced the level and synchrony of DRG neuronal activity, and attenuated mechanical allodynia in the injured hind paw (Fig. 4d–g). Furthermore, after local administration of ATP-hydrolyzing enzyme apyrase (400 U/mL) to the injured sciatic nerve or insulating injured nerve ends by a plastic tube (Supplementary Fig. 3b–f), the activity level and synchrony of DRG neurons dropped significantly, along with the alleviation of mechanical allodynia in SNI mice (Fig. 4d–g). The latter experiment of isolating nerve ends also suggests that ATP released from surrounding tissue rather than injured nerve itself is important for increased DRG synchrony. We also examined neuronal Ca^2+^ response in DRG following local administration of UTP, an agonist in activation of P2Y receptor. We found a moderate increase in the level and synchrony of DRG neuronal activity. Compared to UTP, ATP administration had stronger effect on DRG neuronal activity in SNI mice (Supplementary Fig. 4a, b), suggesting that multiple purinergic receptors may be involved in regulating DRG neuronal activity.

Taken together, the results above suggest that high level of injury-induced ATP release and up-regulation of P2X_3_ receptor expression within several hours after SNI contribute to the initiation of DRG synchrony. Furthermore, ATP released after SNI acts on P2X_3_ receptors of injured nerve fibers to promote neuronal hyperactivity and synchrony in the DRG, as well as animals’ mechanical hypersensitivity in the early phase of neuropathic pain development.

### Peripheral purinergic signaling contributes to SNI-induced cortical plasticity and oscillation

**Fig. 5:**
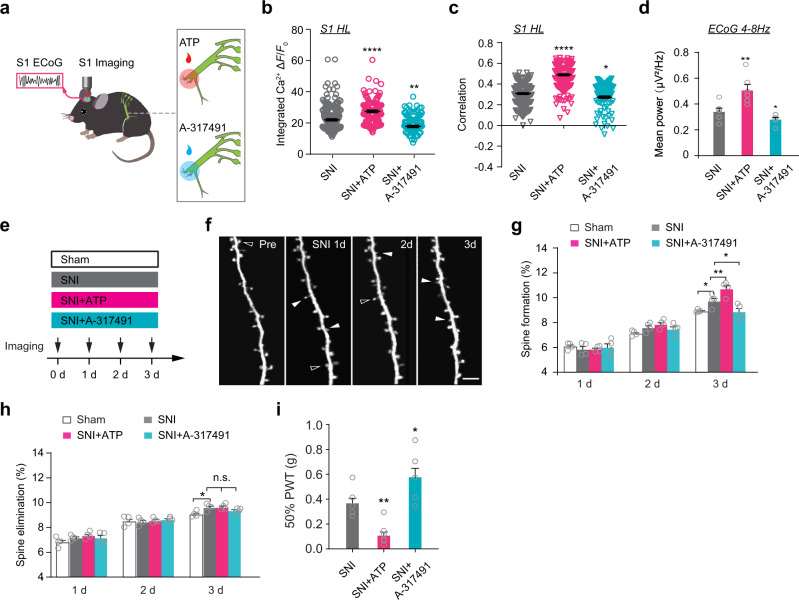
ATP-dependent activity synchrony of DRG neurons is critical for synaptic remodeling in S1. **a** Schematic of Ca^2+^ imaging of L2/3 pyramidal neurons and ECoG recording in S1 of in mice at 3 days after SNI surgery. The inset indicates SNI surgery and local application of ATP or A-317491 to sciatic nerve. **b**, **c** Integrated Ca^2+^ activity (**b**) and correlation coefficient (**c**) of L2/3 pyramidal neurons in S1 and ECoG theta band power (**d**) at 3 days after SNI, plus application of ATP or A-317491 over 3 days after SNI. **e** Schematic of experimental design in panels **f**–**h**. Apical dendritic spines of L5 pyramidal neurons in S1 were imaged daily for 4 days. **f** Representative images of dendritic spines on apical tuft dendrites of layer 5 pyramidal neurons before, 1 day, 2 days, and 3 days after SNI. Filled and empty arrowheads indicate dendritic spines that were formed and eliminated between two consecutive views, respectively. Scale bar: 5 µm. **g**, **h** Percentages of dendritic spine formation (**g**) and elimination (**h**) over time in S1 of sham and SNI mice with or without peripheral treatment of ATP and A-317491. **i** Measurements of PWT in SNI mice with or without repeated ATP/A-317491 treatment over 3 days. Data are expressed as mean  ±  SEM. *P < 0.05, **P < 0.01, ****P < 0.0001. See Table S1 for statistical details. Source data are provided as a Source Data file.

To investigate the impact of peripheral purinergic signaling further, we examined somatic Ca^2+^ activity of L2/3 pyramidal neurons and recorded ECoG in head-fixed mice after reinforcing DRG synchrony by peripheral administration of ATP (Fig. 5a). In this experiment, ATP (10 mM, 3 times per day) was applied to the sciatic nerve for 3 days after SNI surgery. We found that the level and synchrony of Ca^2+^ activity in L2/3 pyramidal neurons (Fig. 5b, c), as well as theta band power in ECoG recording (Fig. 5d, Supplementary Fig. 4a), were significantly elevated after ATP treatment in SNI mice. In contrast, inhibiting DRG activity and synchrony by local administration of the P2X_3_ receptor antagonist A-317491 (200 μM, 6 times a day) for 3 days (Fig. 5a) reduced L2/3 pyramidal neuronal activity and synchrony, as well as cortical theta oscillations (Fig. 5b, d, Supplementary Fig. 4c). Thus, peripheral purinergic signaling after SNI not only enhances DRG activity/synchrony, but also increases cortical synchrony.

Previous studies have shown that SNI increases the rapid remodeling of postsynaptic dendritic spines on apical dendrites of L5 pyramidal neurons in S1, which may underlie long-lasting functional changes in neuropathic pain^50,51^. Consistently, by repeated in vivo imaging of the apical dendrites of L5 pyramidal neurons in *Thy1*-YFP mice with two-photon microscopy^52,53^ (Fig. 5e), we observed significant increases in dendritic spine formation and elimination over 3 days, but not 1 and 2 days after SNI (Fig. 5f, h). Notably, repeated peripheral ATP treatment for 3 days following SNI surgery increased the formation rate of dendritic spines and exacerbated mechanical allodynia, while repeated administration of P2X_3_ receptor antagonist A-317491 reduced the rate of spine formation and relieved mechanical allodynia (Fig. 5g–i).

Taken together, these results underscore that purinergic signaling-dependent DRG neuronal activity/synchrony is critical for enhanced cortical oscillation, synaptic structural remodeling, and mechanical allodynia.

### Increased DRG synchrony is critical for pain-related cortical changes in naive mice

**Fig. 6:**
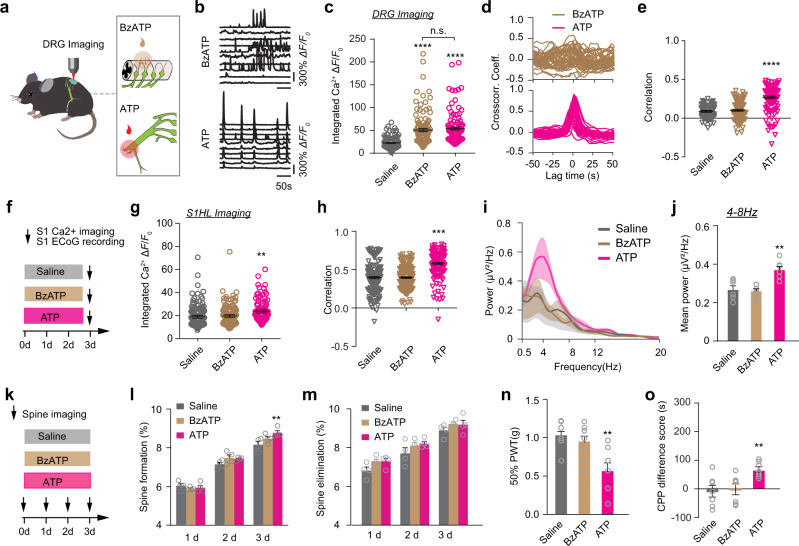
Increased DRG synchrony elicits S1 plasticity and pain-like behavior in naive mice. **a** Schematic of peripheral BzATP or ATP application. **b** Representative fluorescence traces of DRG neurons with application of BzATP or ATP. **c** Integrated Ca^2+^ activity of the DRG neurons induced by local application of 500 μM BzATP was comparable to that induced by peripheral application of 10 mM ATP. **d** Cross-correlograms of the same 10 neurons after BzATP (top panel) and ATP application (bottom panel). **e** Correlation coefficient of DRG neurons before and after ATP or BzATP application in naive mice. **f** Schematic of experimental design. In vivo Ca^2+^ imaging of L2/3 pyramidal neurons and ECoG recording were performed in S1 of naive mice treated with saline, BzATP, or ATP for 3 days. **g**, **h** Integrated Ca^2+^ activity (**g**) and correlation coefficient (**h**) of L2/3 pyramidal neurons in naive mice after repeated saline, BzATP, or ATP treatment. **i**, **j** ECoG power spectra (**i**) and the theta band power (**j**) in naive mice with saline, BzATP or ATP treatment for 3 days. **k** Schematic of in vivo apical spine imaging in S1 of naive mice treated with saline, BzATP or ATP for 3 days. **l**, **m** Percentages of apical dendritic spines of L5 pyramidal neurons formed (**l**) and eliminated (**m**) over 1 day, 2 days, or 3 days in the S1 of naive mice with repeated saline or BzATP or ATP treatment. **n** Measurement of PWT in naive mice with repeated treatment of saline, BzATP or ATP. **o** Preference scores (difference in time spent between lidocaine- and saline-paired compartment) indicative of spontaneous pain were examined in naive mice after repeated treatment of saline, BzATP or ATP for 3 days. Data are expressed as mean  ±  SEM. *P < 0.05, **P < 0.01, ***P < 0.001, ****P < 0.0001. See Table S1 for statistical details. Source data are provided as a Source Data file.

The experiments above show that increasing the level and synchrony of DRG activity induces S1 plasticity and pain behavior after SNI. However, it is unclear whether the level or synchrony of DRG activity is critical for these changes. To address this question, we took advantage of previous findings that activating P2X_7_ receptor of satellite glial cells with BzATP^54,55^ increases neuronal activity in the DRG^56^. For applying BzATP to the DRG, a semicircular coverslip was used to cover 2/3 of DRG with 1/3 of DRG exposed (see methods). Notably, we found that local application of BzATP increased the level but not synchrony of DRG activity (Fig. 6a–d). There was no significant difference in DRG synchrony between pre-treatment condition and BzATP treatment (Fig. 6e). In contrast, local application of ATP to the intact sciatic nerve increased both the level and synchrony of DRG neuronal activity in a dose-dependent manner (Supplementary Fig. 5a). Importantly, while the level of integrated DRG activity elicited by 10 mM ATP in the periphery was comparable to that by 0.5 mM BzATP in the DRG (Fig. 6b, c), ATP induced a significantly higher level of synchrony among DRG neurons than BzATP (Fig. 6d, e; Supplementary Movies 2–4).

The above results provide us an opportunity to investigate whether the level or synchrony of DRG neuronal activity is important for cortical changes in S1. Specifically, we administered either BzATP (0.5 mM, 10 μL) to L3–L5 DRGs or ATP (10 mM, 30 μL) to the sciatic nerve three times a day over 3 days in naive mice. To apply BzATP to L3-5 DRGs, three silicone catheters with 0.5 mm in inner diameter were placed to the posterior edge of the surface of three DRGs, respectively. The catheters were fixed to the vertebrae column to avoid rotation and loosening (see methods). We then performed Ca^2+^ imaging and ECoG recording in S1 (Fig. 6f). As expected, repeated peripheral ATP administration in naive mice increased Ca^2+^ activity level (Fig. 6g) and synchrony (Fig. 6h) in L2/3 pyramidal neurons as well as ECoG power in theta band (Fig. 6i, j, Supplementary Fig. 5b) when compared to the controls with saline treatment. However, repeated BzATP application failed to cause any of these changes (Fig. 6g–j, Supplementary Fig. 5b). Because the level of DRG activity increased after ATP or BzATP treatment but only ATP increased the synchrony of DRG activity, these findings indicate that increased activity synchrony, not level, of DRG neurons is critical for enhanced synchrony of S1 pyramidal neuronal activity and cortical oscillations.

Moreover, repeated imaging of dendritic spines of L5 pyramidal neurons showed that spine formation was significantly increased 3 days after repeated ATP treatment in the sciatic nerve as compared to the controls with saline treatment (Fig. 6k, l). No significant difference was found in spine elimination within 3 days following ATP treatment (Fig. 6m). On the other hand, we found no significant difference in dendritic spine formation or elimination following repeated BzATP treatment when compared to the controls with saline treatment (Fig. 6l, m). Thus, synchronized activation, rather than elevated activity level, of DRG neurons is also important for the reorganization of synaptic connections in S1.

Notably, repeated peripheral ATP treatment also decreased the paw withdrawal threshold substantially in naive mice, whereas BzATP application had no effects on the animals’ paw withdrawal threshold (Fig. 6n). In addition, conditional place preference (CPP) tests showed that mice subjected to repeated peripheral ATP treatment exhibited a preference for the lidocaine-paired chamber as compared to saline controls, indicating the presence of spontaneous ongoing pain in these ATP-treated mice. However, mice subjected to repeated BzATP application spent a similar amount of time in the lidocaine- and vehicle-paired chamber (Fig. 6o). Taken together, these results show that synchronous activity of DRG neurons induced by peripheral ATP application causes mechanical hypersensitivity and persistent spontaneous pain-like behavior. These pain-like behaviors were absent in mice with elevated but non-synchronized DRG neuronal activity induced by BzATP treatment.

## Discussion

Increased cortical synchrony is common in patients as well as animal models of neuropathic pain^3,5,8–10,57,58^. The mechanisms and functional impacts of enhanced cortical oscillations remain unknown. By monitoring neuronal population activity in DRG and S1 in awake, behaving mice, we show that synchronized activity of DRG neurons occurs within hours after injury and 1–2 days prior to the increase in activity synchrony of L2/3/5 pyramidal neurons and low-frequency cortical oscillations in S1. Neuronal activity synchrony in DRG is initiated by axotomized neurons and facilitated by local purinergic signaling at the site of nerve injury. Importantly, the synchrony, not level, of DRG neuronal activity is critical for cortical plasticity, mechanical hypersensitivity, and spontaneous pain-like behavior. These findings reveal the important role of the synchronized activity of DRG neurons in enhanced cortical oscillations and neuronal plasticity in S1 in the development of neuropathic pain.

The altered activity of DRG neurons is involved in the development of neuropathic pain^59,60^. However, due to technical difficulties, the DRG neuronal activity has not been investigated in awake animals under pain conditions. Our discovery of DRG neuronal synchrony after SNI benefits from the recent technological advancement of DRG imaging in awake-behaving mice^25^, as DRG neuronal activity is largely blocked in animals under anesthesia^38,61^. In addition to SNI mice, we found that the level and synchrony of DRG neuronal activity were also significantly higher in mice after plantar Complete Freund’s Adjuvant (CFA) or formalin injection as compared to pre-injection (Supplementary Fig. 6a–f), suggesting that synchronized activity of DRG neurons may be a common feature in the development of chronic pain caused by nerve injury or inflammation. Of note, Although GCaMP6s produces large fluorescence transients due to somatic calcium increase triggered by a single action potential^62^, its slow dynamics make it difficult to resolve the number of action potentials. Therefore, our in vivo imaging data were not able to decode the firing rates of these neurons.

Spontaneous and synchronized neuronal activity is thought to be important for the development of sensory circuits^63–65^. Indeed, developing retinal ganglion neurons exhibit spontaneous co-activity in the form of retinal waves before eye opening^66^. In the developing cochlea, spiral ganglion neurons are also spontaneously co-active before the hearing onset^65^. This synchronized neuronal activity from retina/peripheral spiral ganglia is critical for establishing and maintaining tonotopic maps in visual/auditory pathways^67,68^, likely through Hebbian plasticity mechanisms such that synchronously active neurons tend to be wired together with the same target cells^64^. Zheng et al.^69^ showed that spontaneous DRG cluster firing mediated spontaneous pain, and abnormal sprouting of sympathetic fibers into DRG may be involved in this synchronized activation. Following pain induction, there is an increase in synchronized neuronal activity in S1, and artificially increasing neuronal activity and synchrony reduces pain thresholds^70^. It is possible that after nerve injury, synchronized neuronal activation in DRG and S1 exerts a similarly profound impact on neuronal circuits, leading to heightened cortical plasticity and emotional-behavioral expressions of pain. In support, our results show that increasing or decreasing peripheral DRG neuronal synchrony drastically enhances or reduces pyramidal neuronal activity synchrony and dendritic spine turnover, theta band power in S1, as well as pain sensitivity respectively, underscoring that synchronized peripheral inputs are the major driving force for cortical changes during the development of neuropathic pain.

Our data suggest that injured afferents become co-active as early as 3 hours post SNI surgery, and that enhanced ATP signaling at the traumatic zone is important for the progressive increase in DRG activity synchrony. In the cochlear, the synchronized activity of spiral ganglion neurons is triggered by spontaneously released ATP from surrounding glia-like supporting cells^65^, and amplified by NMDA receptor-dependent excitability, as pharmacologically blocking NMDA receptors reduces the number of spiral ganglion cells that participate in each synchronous event^71^. Gap junctions and NMDA receptors were also reported to be involved in the synchronous activation of neural ensembles across sensory systems^72–75^. Whether mechanisms involving gap junctions and glial cells underlie the increased DRG synchrony in neuropathic pain development merits further investigation.

Although our results revealed the causal relationship between DRG synchrony, pyramidal neuron synchrony and 4–8 Hz oscillations in S1, how synchronized DRG activity enhance S1 oscillations over extended periods of time is complicated and likely involves progressive changes of multiple cell types (glial^22,32,76^, inhibitory neurons^20,22^ and pyramidal neurons^19,20^) at molecular and cellular levels. While our study identifies DRG synchronized activity as the driving force for cortical changes and increased S1 oscillations, the mechanistic links between DRG synchrony and S1 oscillations remain to be established in the future. In the present study, we employed a drug delivery approach for DRG synchrony and asynchrony induction. Since the expression of opsins in DRG neurons combined with transdermal light illumination could successfully modulate DRG neuronal activity and pain intensity^77^, such an optogenetic approach may be more precise for DRG synchrony induction and mechanistic studies in the future.

Predicting pain intensity objectively in patients with chronic pain is currently challenging but important for appropriate pain treatment^22^. Previous studies from patients and animals have reported changes in electrical activity over wide frequency ranges under pain condition^3–5,22^. Our study implicates that among diverse oscillatory rhythms, low-frequency cortical oscillations, particularly at the theta range, was significantly enhanced during neuropathic pain development. This is consistent with observations in human subjects that enhanced theta oscillatory occurs in pain states^8,9^. On the other hand, Tan et al.^22^ reported that gamma oscillations, but not other rhythms, are specifically strengthened in the S1 cortex of mice upon noxious stimulation. Gamma oscillatory has also been linked to the pain states elicited by noxious stimuli in human subjects^78–80^. It is important to note that in our study, low-frequency cortical oscillations are associated with spontaneous electrical activity rather than stimulus-induced oscillatory activity as reported in other studies^22^. Our findings of DRG neuronal synchrony provide novel insights into the origin and impact of increased synchronization of neuronal networks during pain development. They suggest that DRG synchrony and cortical oscilations could be potential biomarker for developing analgesics. Furthermore, suppressing peripheral activity synchronization could be a new strategy for chronic pain treatment.

## Methods

### Experimental animals

Transgenic mice expressing GCaMP6s in a subset of afferent neurons in DRGs and in layer 2/3 pyramidal neurons, *Thy1*-GCaMP6 slow line 3, were used. In DRG, GCaMP6 expression was observed in a subset of primary sensory neurons^25,81^. Transgenic mice expressing YFP in layer five pyramidal neurons, *Thy1*-H-YFP line were obtained from the Jackson Laboratory. Wildtype C57BL6J mice were obtained from the Jackson Laboratory. Ten to 14-week-old animals of both sexes were used for all experiments. Mice were group-housed in rooms with temperature control of 25 Celsius degree and humidity ~50% on a 12-h light-dark cycle and were randomly assigned to different treatment groups. IACUC Institutional Review Boards at Shenzhen Bay Laboratory, New York University, and Columbia University approved all animal procedures. Experiments were conducted in accordance with the National Institute of Health Guide for the Care and Use of Laboratory Animals.

### Animal models

Spared SNI of the sciatic nerve or sham operation was performed in *Thy1*-GCaMP6s mice^34^. In brief, mice were anesthetized with 3.5% isoflurane in air. Anesthesia was maintained with 1.2% isoflurane in air. A 0.5 cm-long incision in the left thigh was made to expose the sciatic nerve and its three branches. The common peroneal nerve and tibial nerve were ligated and axotomized by removing a 2–4 mm piece of each distal nerve stump. The sural nerve was kept intact. For sham surgery, the sciatic nerve was exposed but not ligated or cut. In the case of tibial nerve injury (Fig. 3a), tibial nerve was ligated and cut, while in the case of spared tibial nerve injury (Fig. 3d), common peroneal nerve and sural nerve were ligated and cut.

Inflammatory pain was induced by subcutaneous CFA^82^ or formalin injection^83^. Specifically, 10 µL CFA (mixed with saline in a 1:1 ratio) or 10 µL formalin (2%) solution was injected under the plantar surface of the left hindpaw with a 30-gauge needle.

### In vivo DRG Ca^2+^ imaging

In vivo imaging of L4 DRG in awake mice was performed according to method described recently^25^. In brief, mice were placed in a 2.9-cm-diameter transparent plastic cylinder to attenuate motion artifacts. The cylinder was then mounted onto a heavy metal base. Before imaging, mice were habituated for at least 30 min. *Thy1*-GCaMP6s mice were used for Ca^2+^ imaging of afferent sensory somas in the DRG. Imaging was performed using a Bruker two-photon system equipped with a DeepSee Ti:sapphire laser (Spectra Physics) tuned to 920 nm. The average laser power on the sample was ~20–30 mW. Images were collected at frame rates of 1.3–1.7 Hz at a resolution of 512 × 512 pixels using a ×25 objective (NA = 1.05; Olympus America) immersed in artificial cerebrospinal fluid (ACSF) and with a 1× digital zoom. Image acquisition was performed using Bruker PrairieView software.

### In vivo Ca^2+^ imaging in S1 HL region

Ca^2+^ imaging in L2/3 of S1 hindlimb (HL) was performed in awake mice through a thinned skull window^53^. In brief, mice were anesthetized with an intraperitoneal injection of 100 mg/kg ketamine and 15 mg/kg xylazine (KX), and the head was shaved and the skull surface was exposed with a midline scalp incision. After removing the periosteum tissue, a head holder was attached to the skull surface with cyanoacrylate glue (Loctite 495) and dental acrylic cement. After identifying the hindlimb region of S1 in the contralateral hemisphere on the basis of stereotaxic coordinates (0.5 mm posterior and 1.5 mm lateral to the bregma), a high-speed micro-drill was used to thin a circular area (~0.2 mm in diameter) to a thickness of ~20 μm. The exposed skull was covered with a thin layer of Kwik-Sil® silicone elastomer. Imaging was performed 24 hours after surgery in well-habituated mice. Before imaging, the skull was immersed in ACSF and the head-restrained animal was then placed on the stage of a two-photon laser scanning microscopy. In some experiments (Fig. 1f), somatic activity of L2/3 pyramidal neurons was repeatedly imaged over a period of 3 days through a reinforced thinned skull window^84^.

### Two-photon imaging of dendritic spines in S1 HL region

The surgical procedure for chronic transcranial two-photon imaging has been described previously^52^. While the animal was under deep KX anesthesia, the skull surface was exposed with a midline scalp incision, and a 0.2-mm-diameter skull region over the S1 HL region was identified based on stereotaxic coordinates. A custom-made, stainless steel plate was glued to the skull with a central opening over the cortical region of interest. Apical tuft dendrites of layer 5 pyramidal neurons in S1 were repeatedly imaged over a period of 3 days. Image stacks of dendritic segments located in the superficial cortical layer were obtained using a Bruker two-photon microscope with the laser tuned to 920 nm and with a ×25 objective (NA = 1.05; Olympus America) immersed in ACSF. A 3× digital zoom was used to yield high-magnification images (169 μm × 169 μm; 1024 pixel × 1024 pixel, 0.75 μm Z-step size) suitable for quantification of dendritic spines. For multiple imaging, the above procedure was repeated and the localization of the same region was facilitated by low-magnification image stack at 1× digital zoom (508.43 μm × 508.43 μm; 1024 pixel × 1024 pixel) and with reference to vascular landmarks under the thinned skull area. The animal was head-restrained during image acquisition, which lasted ~30 minutes. The scalp incision was immediately sutured after each imaging session.

### Bioluminescence imaging of ATP

ATP released from the sciatic nerve and surrounding tissue was imaged by an IVIS Spectrum (Caliper Life Sciences, MA). d-Luciferase/luciferin solution [Thermo Fisher, A22066, D-Luciferase (0.5 mg/mL), luciferin (3 mg/mL)] was prepared with 1× DPBS (Dulbecco’s phosphate-buffered saline) solution. A volume of 100 μL was administered systemically via retro-orbital injection when the mice were maintained under isoflurane anesthesia. Bioluminescence images were taken using an IVIS Spectrum with 60 second exposure time, medium binning (8), and f/stop 1. Light outputs were quantified using Living Image 4.5.5 (Caliper Life Sciences, Alameda, CA).

### Surgical procedure for in vivo DRG imaging

Surgical procedure for DRG window implantation was performed as described recently^25^. In brief, mice were deeply anesthetized with KX and placed under a stereomicroscope (Leica MZ12.5; Leica Microsystems). A 1-cm-long incision was made in the dorsal skin at the L3–L5 level of the spine, and the muscles along the lateral aspects of L3–L5 were detached and then retracted with a custom-made metal sheet. To expose the left L4 DRG, we aligned the long bracket of the imaging device with the left side of L4–L5 vertebrae and short bracket with the right side, and then registered the two brackets with a locking screw. After trimming articular processes around the DRG, a thin layer of Kwik-Sil® silicone elastomer and a round coverslip was placed on top. For applying drugs to the DRG, a semicircular coverslip was used to cover 2/3 of DRG with 1/3 of DRG and 1-mm-long spinal nerve exposed. The exposed nerve tissue was protected with 1% agarose. The coverslip was secured to the vertebrae mount by cyanoacrylate and dental acrylic. Throughout the surgical procedure and recovery, the animal’s body temperature was maintained at ~37 ^o^C.

### Electrode implantation and ECoG recording in S1 HL region

Under KX anesthesia, mice were head-fixed with a stereotaxic apparatus. A small skin incision was used to expose the skull and a small area of skull (~1 mm in diameter) over S1 contralateral to the left hind paw (0.5 mm posterior to and 1.5 mm lateral from bregma) was thinned with a high-speed drill and carefully removed with forceps. Two electrodes for ECoG recording were made by soldering one end of an epoxy-coated silver wire (0.003 inch in diameter, A-M Systems) to a female miniature multichannel connector. The electrodes were inserted above the dura mater of the cortex with the tips separated from each other by ~500 μm. The electrodes were fixed by cyanoacrylate-based glue and dental cement. When simultaneously performing Ca^2+^ imaging of L2/3 pyramidal neurons in S1, two ECoG electrodes were placed in the area close to S1 (2.0 mm posterior and 1.5 mm lateral to the bregma). Two copper wires were placed in the nuchal muscles for electromyographic (EMG) recordings. Recording sessions began 3-5 days after electrode implants. Connectors from ECoG electrode were tethered to preamplifier headstage leading to a multichannel amplifier. Simultaneously recorded ECoG and EMG signals were amplified 500 times (BL-420F Data acquisition and analysis system, Techman Software Co.LTD, Chengdu, China) and ECoG data were analyzed using custom code in MATLAB (Mathworks, R2017b, Natick, MA). The code, a demo, and instructions for analysis are provided in Supplementary Software 1.

Fast Fourier transformation was performed to convert ECoG waveform from the time domain to the frequency domain, yielding power spectra density using 11.46 sec (Simultaneously record Ca^2+^ activity of L2/3 pyramidal neurons in S1, 11.46 sec = 10 imaging frames) or 10 sec time intervals during awake resting state (no noticeable movement and sleep) in each animal. Relative power values were generated at a sampling frequency of 5 kHz between 0.5 and 40 Hz. Mean power spectra density was calculated between 0.5–4 (delta band), 4–8 (theta band), and 8–12 Hz (alpha band).

### DiI labeling

To label the tibial branch-specific-neurons in the L4 DRG, the retrograde nerve tracer DiI (D282, Thermo Fisher, 0.5% in anhydrous alcohol; 0.2 μL) was injected (Picospritzer III; 15 p.s.i., 12 ms, 0.8 Hz) into the trunk of tibial nerve using a glass microelectrode over a period of 8–10 min.

### Sciatic nerve isolation in SNI mice

Mice were anesthetized with isoflurane and a 0.8-cm-long incision was made on the dorsal skin of the left thigh. The muscles attached to the femur bone were detached and distal 2/3 of the femur was exposed. A high-speed drill was used to create two 0.5-mm-diameter holes on the distal 1/3 and 1/4 of the femur with. With the aid of these two holes, a plastic bottle (7-mm-long; 3-mm-inner diameter of the body; 1-mm- inner diameter of the neck) without lid and bottom was tied to the femur using the 6–0 suture (Supplementary Fig. 3a, b). The distal and proximal ends of the bottle were sealed with silicone elastomer to prevent connective tissue from invading, and then the incision was closed.

To minimize the potential effects of surgery-related inflammation, SNI surgery was performed 7 days after bottle implantation (Supplementary Fig. 3c). Three days after SNI surgery, DRG imaging was first performed before nerve isolation. Thereafter, the proximal nerve end was pulled into the bottle through a suture attached to the nerve end, and the nerve was overhang inside the bottle without tension and compression at the bottleneck (Supplementary Fig. 3d). The bottle was subsequently filled with 0.9% saline and sealed with silicone elastomer. DRG imaging was performed 6–8 hours after nerve isolation so as to minimize nerve irritation (Supplementary Fig. 3e).

### Catheter implantation to the surface of DRG

A 1.0-cm-long incision was made in the dorsal skin along the L3–L5 of the spine under deep KX anesthesia. Muscles and ligaments attached to the lateral aspects of the three vertebrae were detached using surgical scissors. The connective tissue over the DRGs was removed and three silicone catheters with 0.5 mm in inner diameter were inserted into the posterior edge of the surface of L3–L5 DRGs, respectively, to avoid injury to DRGs. The catheters were fixed to the vertebrae column using 8-0 suture. At least two points of each catheter were fixed to the ligamenta interspinalia and spinous processes to avoid rotation and loosening. Guidewire was inserted in the silicone catheters to prevent potential blockage by connective tissue or blood clots, pulled out before each drug delivery, and reinserted in the catheters afterwards. At the end of the behavioral experiments, we confirmed that the tip of the intrathecal catheter placement was near the DRG surface.

### Drug delivery

ATP (A2383, Sigma-Aldrich), A-317491 (A2927, Sigma-Aldrich), or apyrase (A6535, Sigma-Aldrich) were locally applied with 100 μL microinjector to the sciatic nerve at the incision site. Entry point was located medial to linea aspera (third trochanter) of the fumer that was easily identified. ATP (5–30 mM), A-317491 (200 μM) and apyrase (400 U/mL) were dissolved directly in 0.9% saline solution to final concentrations. ATP and A-317491 were applied once (30 μL), and apyrase was applied four times within 30 minutes (50 μL every 10 min). To examine the effect of BzATP on DRG neuronal activity by in vivo DRG imaging, distal 1/3 of DRG were exposed for drug delivery. BzATP (ab120444, Abcam, 0.5 mM, 30 μL) was locally applied to distal 1/3 of DRG during in vivo imaging (See Surgical procedure for in vivo DRG imaging). To examine the behavioral effects of DRG asynchrony induction, BzATP (0.5 mM, 10 μL) was delivered via intrathecally implanted catheter, with three tips placed adjacent to the left lumbar 3–5 DRGs respectively.

### Behavioral tests

For mechanical allodynia test, mice were individually placed into transparent plastic boxes (10 cm × 7 cm × 7 cm) over a mesh table and habituated for at least 30 min prior to testing procedures. Dixon’s up and down method was employed to measure the animals’ paw withdrawal threshold^85^. In short, a series of von Frey fibers (0.008, 0.02, 0.07, 0.16, 0.4, 0.6, 1.0, 1.4, 2.0, and 4.0 g) were presented in a consecutive ascending order. In the absence of paw withdrawal response, the next stronger stimulus was presented; in the event of paw withdrawal, the next weaker stimulus was chosen. After the response threshold was first crossed, 6 data points were counted, at which time the 2 responses straddling the threshold were retrospectively designated as the first 2 responses of the series of 6. 50% response threshold was calculated as: 50% g threshold = (10 ^[*X*f^ ^+^ ^*κδ*]^)/10,000 where *X*_f_ = value (in log units) of the final von Frey hair used; *κ* = tabular value for the pattern of positive/negative responses^86^; and *δ* = mean difference (in log units) between stimuli (here, 0.2699).

The CPP test was performed as previously described^87^. Briefly, the test apparatus consisted of a shuttle-box that was divided into two compartments of the same size (32 cm × 15 cm × 25 cm; Varese, Italy) by a guillotine door (4 cm wide × 6 cm high). Each compartment had different visual and textured cues (e.g. wall patterns, floor patterns, and texture). Mice were habituated to the apparatus for 10 min for 3 consecutive days prior to conditioning. In the pre-conditioning phase (day 0), animals were placed in the apparatus and the door was removed to allow animals free access to both chambers for 10 min. The time spent in each compartment was recorded. The animals showing strong unconditioned preference (>400 s) were discarded. On day 1, mice were randomly assigned for SNI or sham surgery (Supplementary Fig. 1b), or mice were randomly assigned for routine injection of ATP to the sciatic nerve or BzATP to L3–L5 DRGs (Fig. 6o). During conditioning (days 1–3), mice were injected with vehicle control (5 μL saline, i.th.) in the morning and immediately confined to the paired compartment for 30 min; ~6 hours later, the mice were injected with lidocaine (0.04% in 5 μL saline, i.th.), and immediately placed in the other compartment for 30 min. Chambers were counter-balanced assigned for drug pairing in all the test mice. CPP test was carried out on day 4. Mice were placed in the apparatus and allowed free access to both compartments for 10 min. The time spent in each compartment was recorded for each animal.

### Western blot assay

A length of 0.5 mm of the injured nerve trunk proximal to DRG was collected for Western blot assay. Protein samples were prepared and loaded onto a 4–15% stacking/7.5% separating SDS-polyacrylamide gel (Bio-Rad, 345-0032) as reported previously^88^. The proteins on the gel were then electrophoretically transferred onto a polyvinylidene difluoride membrane (Bio-Rad, 162-0233). Membranes were blocked with 3% nonfat milk in Tris-buffered saline containing 0.1% Tween-20 (pH = 7.40) for 1 hour at room temperature. Membranes were first incubated with rabbit anti-P2X_3_ (Sigma, AB5895, 1:500) on an orbital shaker in 4 °C cold room overnight, then further incubated with goat anti-rabbit horseradish peroxidase-conjugated secondary antibody (1:3000; Jackson ImmunoResearch) for 2 hours at room temperature and visualized by western peroxide reagent and luminol/enhancer reagent (Clarity Western ECL Substrate, Bio-Rad). Images were generated with the ChemiDoc XRS System with Image Lab software (Bio-Rad). Intensities of protein bands were quantified using the Image Lab software. Values of P2X_3_ proteins were normalized to those of β-actin.

### Data analysis

To analyze Ca^2+^ activity, regions of interest (ROIs) corresponding to visually identifiable somas of DRG neurons were selected for quantification at the depth of 30–200 μm below the surface of DRG. ROIs for L2/3 somas in S1 were selected at the depth of 200–250 µm below the pial surface. The fluorescence in each soma was measured with NIH ImageJ by averaging all pixels within the ROI covering the soma. The Δ*F*/*F*_0_ was calculated as Δ*F*/*F*_0_ = (*F*−*F*_0_)/*F*_0_, where *F*_0_ is the baseline fluorescence signal averaged over a 2-s period corresponding to the lowest fluorescence signal over the 2.5-min recording period. Integrated Ca^2+^ activity is the sum of Δ*F*/*F*_0_ over 2.5 min. Active somas were defined with the criteria that Ca^2+^ transient was beyond the threshold of three times the standard deviation of the baseline.

We calculated the cross-correlation for all cell pairs that were imaged simultaneously. Only cells displaying active Ca^2+^ transients were included in the analysis. We calculated the cross-correlation over the time course of 150 seconds. To determine the significance of the correlation at zero time lag we used bootstrap shuffling. Specifically, we shuffled the activity of one cell by a random amount relative to the activity of the paired cell and then re-calculated the cross-correlation for the shuffled data. We repeated this process 500 times and calculated the number of times the value of the cross-correlation at zero time lag for the shuffled data was higher than the original data. If the value of the shuffled data was higher than the original data in <5% of the shuffles, we considered the correlation between the cell pair to be significant. Data analysis was performed using toolboxes and custom code in MATLAB.

The procedure for quantifying spine dynamics has been described in our previous studies^52,53^. Briefly, image stacks were analyzed using NIH Image J software. For each dendritic segment analyzed, filopodia were identified as long, thin protrusions with a ratio of head diameter to neck diameter <1.2:1 and a ratio of length to neck diameter >3:1. The remaining protrusions were classified as spines. Spines were considered the same between views if their positions remained the same distance from relative adjacent landmarks. Spines were considered different if they were >0.7 μm away from their expected positions based on the first view. More than 150 spines were analyzed from each animal. The minimum length of dendritic branches included in the analysis was over 30 µm. The formation or elimination rates of spines were measured as the number of spines formed or eliminated divided by the number of spines existing in the first view.

### Statistical analysis

Summary data are presented as means ± sem. Sample sizes were chosen to ensure adequate power with the statistical tests while minimizing the number of animals used in compliance with ethical guidelines. No samples or animals that were successfully imaged or measured were excluded from the analysis. The variance was similar between the groups that were statistically compared. Tests for differences between two populations were performed using unpaired or paired *t* test. One-way or two-way analysis of variance followed by Dunnett’s or Tukey’s test was also used to compare the significance between various groups. Significant levels were set at *P* ≤ 0.05. All statistical analyses were performed using GraphPad Prism.

### Reporting summary

Further information on research design is available in the Nature Portfolio Reporting Summary linked to this article.

## Supplementary information

Supplementary Information

Description of Additional Supplementary Files

Supplementary Movie 1

Supplementary Movie 2

Supplementary Movie 3

Supplementary Movie 4

Supplementary Software 1

Reporting Summary

## Data Availability

All the data generated in this study are provided in the Supplementary Information and Source Data file. Source data are provided with this paper.

## Source data

Source Data

## Code availability

Codes and demo files are provided in the Supplementary Information.
