# Supplementary material for: Synchronized activity of sensory neurons initiates cortical synchrony in a model of neuropathic pain
Source: Nat Commun. 2023 Feb 8;14:689. doi: 10.1038/s41467-023-36093-z (PMC9908980; doi:10.1038/s41467-023-36093-z)
Supplement: Supplementary file 1 — Supplementary Information [file 41467_2023_36093_MOESM1_ESM.pdf]

Supplementary Information

Synchronized activity of sensory neurons initiates cortical synchrony in a model of neuropathic pain

Chen et al.

Supplementary figures

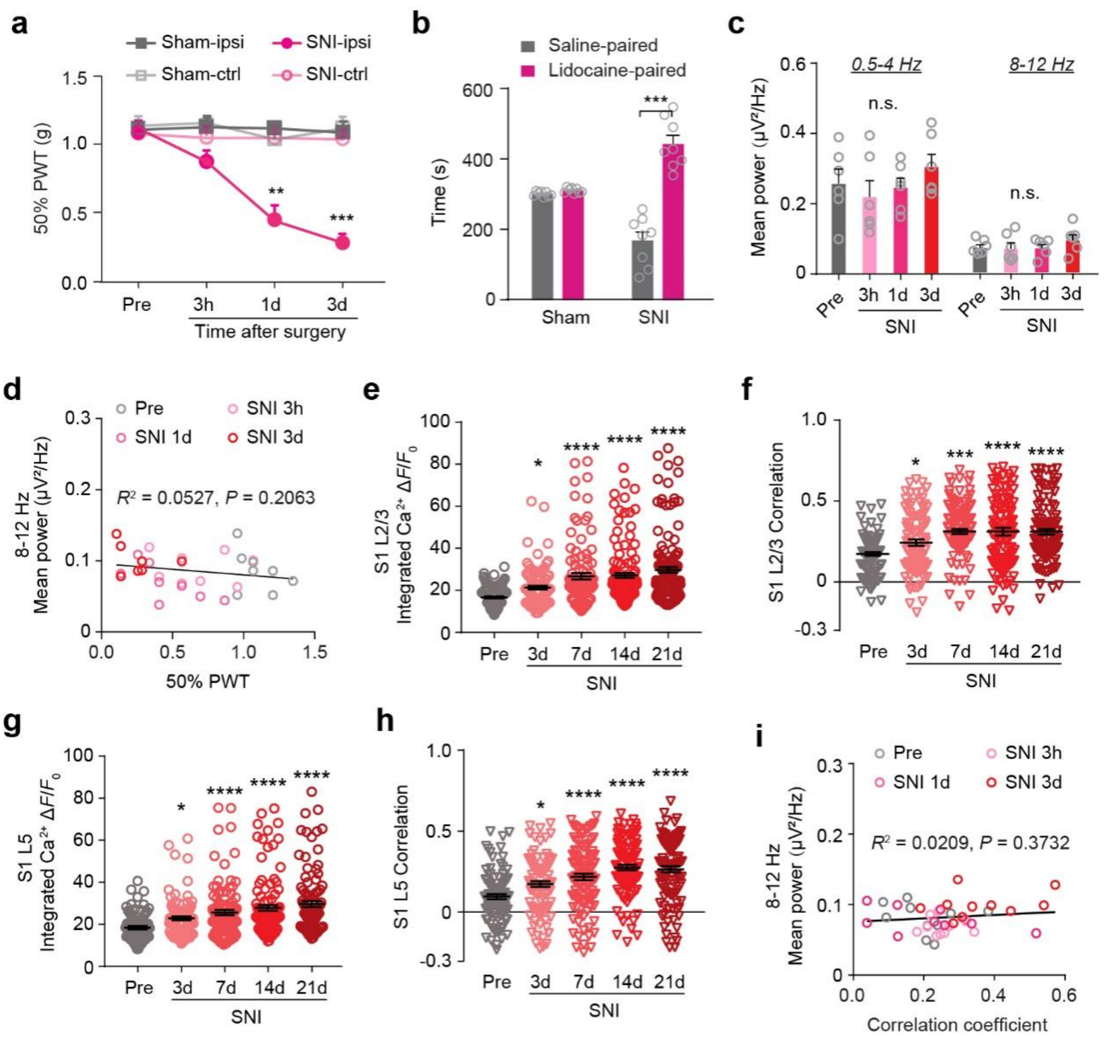

Supplementary Fig. 1| Pain behavior and ECoG power in S1 hindlimb region in SNI mice. (**a**) PWT on ipsilateral and contralateral sides before and after SNI and sham operations. n = 7 mice per group. (**b**) Time spent in saline- or lidocaine- paired compartment in sham or SNI mice. Preference for lidocaine-paired side indicates the presence of spontaneous ongoing pain. n = 8 mice per group. (**c**) The mean power in delta (0.5–4 Hz) and alpha (8–12 Hz) bands before, 3 hours, 1 day and 3 days after SNI. n = 8 mice. (**d**) The mean power in alpha band has no correlation with the animals' paw withdrawal threshold. n = 8 mice. (**e-f**) The integrated Ca^{2+} activity (**e**) and correlation coefficient (**f**) of L2/3 pyramidal neurons in S1 before and 3 days, 7

days,14 days and 21 days after SNI surgery. n = 138 neurons from 8 mice per group. (**g-h**) The integrated Ca^{2+} activity (**g**) and correlation coefficient (**h**) of L5 pyramidal neurons in S1 before and 3 days, 7 days,14 days and 21 days after SNI surgery. n = 162 neurons from 8 mice per group. **(i**) The mean power in alpha band has no correlation with the synchrony level of L2/3 pyramidal neurons in S1. n =8 mice. Data are expressed as mean ± SEM. Two-way ANOVA followed by Dunnett's multiple comparisons test was used in **a**, **b**; One-way ANOVA followed by Sidak's multiple comparisons test was used in **c**, **e**-**h**. ** $P < 0.01$, *** $P < 0.001$.

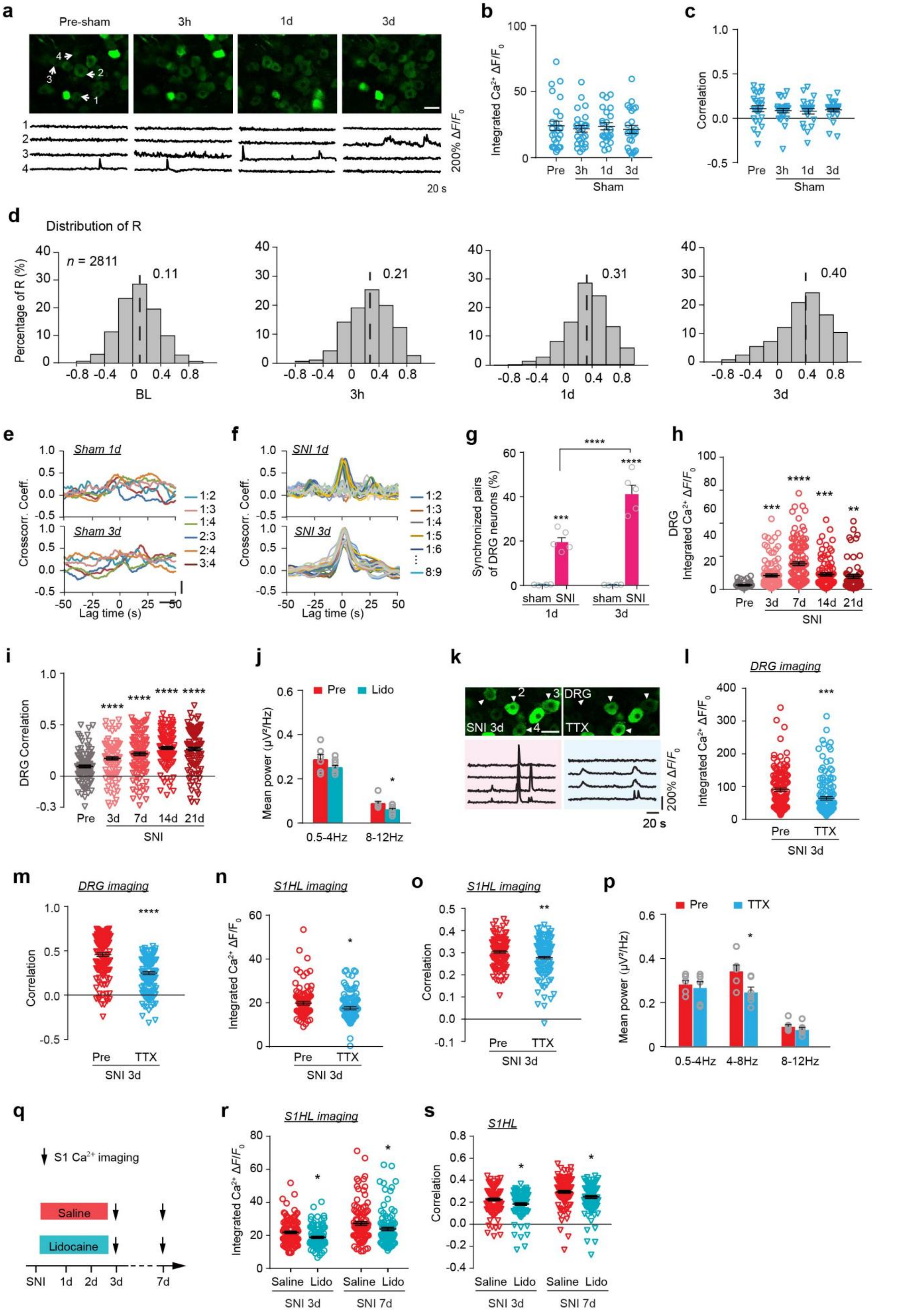
a
Pre-sham
3h
1d
3d
200% ΔF/F0
20 s
b
Integrated Ca2+ ΔF/F0
Pre
3h
1d
3d
Sham
c
Correlation
Pre
3h
1d
3d
Sham
d
Distribution of R
n = 2811
0.11
0.21
0.31
0.40
Percentage of R (%)
BL
3h
1d
3d
e
Sham 1d
Sham 3d
Crosscorr. Coeff.
Lag time (s)
1:2
1:3
1:4
2:3
2:4
3:4
f
SNI 1d
SNI 3d
Crosscorr. Coeff.
Lag time (s)
1:2
1:3
1:4
1:5
1:6
8:9
g
Synchronized pairs of DRG neurons (%)
sham
SNI
1d
3d
h
DRG Integrated Ca2+ ΔF/F0
Pre
3d
7d
14d
21d
SNI
i
DRG Correlation
Pre
3d
7d
14d
21d
SNI
j
Pre
Lido
Mean power (μV2/Hz)
0.5-4Hz
8-12Hz
k
SNI 3d
DRG
TTX
200% ΔF/F0
20 s
l
DRG imaging
Integrated Ca2+ ΔF/F0
Pre
TTX
SNI 3d
m
DRG imaging
Correlation
Pre
TTX
SNI 3d
n
S1HL imaging
Integrated Ca2+ ΔF/F0
Pre
TTX
SNI 3d
o
S1HL imaging
Correlation
Pre
TTX
SNI 3d
p
Pre
TTX
Mean power (μV2/Hz)
0.5-4Hz
4-8Hz
8-12Hz
q
S1 Ca2+ imaging
Saline
Lidocaine
SNI
1d
2d
3d
7d
r
S1HL imaging
Integrated Ca2+ ΔF/F0
Saline
Lido
SNI 3d
SNI 7d
s
S1HL
Correlation
Saline
Lido
SNI 3d
SNI 7d

Supplementary Fig. 2| DRG activity and synchrony contributes to S1 plasticity. (a) Representative two-photon images and fluorescence traces of L4 DRG sensory neurons before, 3 hours, 1 day and 3 days after sham surgery. Arrows point to cells measured. Scale, 50 μm. (**b-c**) The integrated Ca^{2+} activity (**b**) and correlation coefficient (**c**) of DRG neurons before and after sham surgery remain stable. n = 46 neurons from 2 mice per group. **(d)** Distribution of correlation coefficient R of DRG neurons before, 3 hours, 1 day and 3 days after SNI. (**e**) Representative cross-correlograms for the pairwise comparisons of 4 active neurons 1 day and 3 days after sham surgery in **Supplementary Fig. 2a**. (**f**) Cross-correlograms for the pairwise comparisons of 9 active neurons 1 day and 3 days after SNI surgery in **Fig. 2b**. (**g**) The percentage of synchronized pairs of active DRG neurons 1 day and 3 days after sham or SNI surgery. (**h-i**) The integrated Ca^{2+} activity (**h**) and correlation coefficient (**i**) of DRG neurons before and 3 days, 7 days,14 days and 21 days after SNI surgery. n = 78 neurons from 4 mice in sham group and n = 118 neurons from 5 mice in SNI group. (**j**) S1 ECoG power in delta and alpha bands before and after peripheral lidocaine application. n = 8 mice. (**k**) Representative two-photon images and fluorescence traces of DRG sensory neurons before and after local application of TTX to sciatic nerve 3 days after SNI surgery. Arrows point to cells measured. Scale, 50 μm. **(l-m)** The integrated Ca^{2+} activity (**l**) and correlation coefficient (**m**) of DRG neurons before and after local application of TTX. n = 124 neurons from 4 mice. **(n-o)** The integrated Ca^{2+} activity (**n**) and correlation coefficient (**o**) of L2/3 pyramidal neurons in S1 before and after peripheral TTX application at 3 days after SNI surgery. (**p**) The mean ECoG power in delta, theta and alpha bands before and after TTX application at 3 days after SNI. n = 139 neurons from 4 mice. (**q**) Schematic of experimental design in panels **r-s**. *In vivo* Ca^{2+} imaging of L2/3 pyramidal neurons in S1 was performed at 3 days and 7 days in SNI mice

treated with saline or lidocaine for 3 days after SNI. (**r-s**) The integrated Ca^{2+} activity (**r**) and correlation coefficient (**s**) of L2/3 pyramidal neurons in S1 at 3 days and 7 days in SNI mice after repeated saline or lidocaine treatment for 3 days after SNI. n = 147 neurons from 4 mice. One-way ANOVA followed by Sidak's multiple comparisons test was used in **b**, **c**, **h and i**; Two-way ANOVA followed by Dunnett's multiple comparisons test was used in **g**; two-tail paired t test was used in **j-p**, **r-s**. Data are expressed as mean ± SEM. * $P < 0.05$, ** $P < 0.01$, *** $P < 0.001$, **** $P < 0.0001$. $^{####}$ $P < 0.0001$.

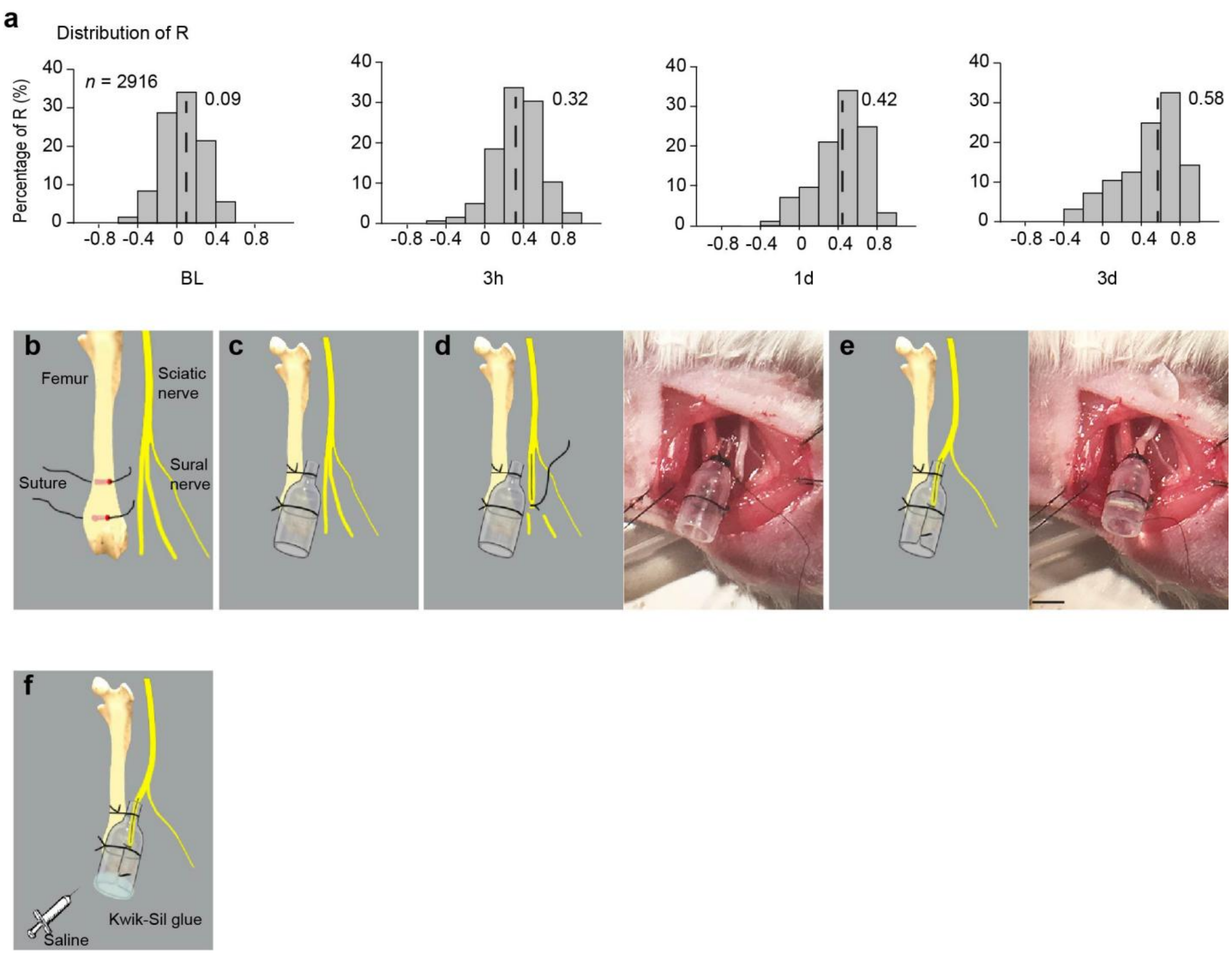

Supplementary Fig. 3| (a) Distribution of correlation coefficient R of axotomized DRG neurons before, 3 hours, 1 day and 3 days after SNI. n = 56 neurons from 3 mice. (**b-f**) Isolation of the injured nerve end in SNI mice. **(b**) Schematic showing two 0.5-mm-diameter holes drilled on the distal 1/3 and 1/4 of the femur. Two 6-0 sutures passed through the two holes in the femur. (**c**) A plastic bottle without lid and bottom was tied to the femur with the aid of the two sutures. (**d**) SNI surgery was performed 5–7 days after bottle immobilization. (**e**) The nerve end was pulled into the bottle through a suture attached to the nerve end. The distal end of suture was fixed to the wall of the bottle and the nerve overhang inside the bottle. (**f**) The bottle was sealed with silicone elastomer and filled with 0.9% saline.

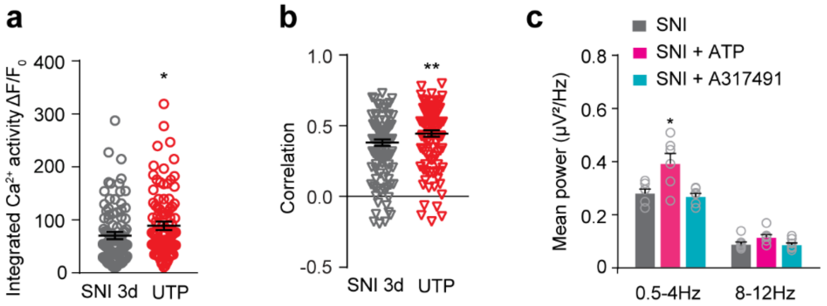

Supplementary Fig. 4| (a-b) The integrated Ca^{2+} activity (**a**) and correlation coefficient (**b**) of DRG neurons before and after local application of UTP at 3 days after SNI. n = 176 neurons from 4 mice. (**c**) ECoG delta and alpha band power at 3 days after SNI, plus application of ATP or A-317491 over 3 days after SNI. n = 6 mice per group. Two-tail paired t test was used in **a**, **b**; One way ANOVA followed by Dunnett's multiple comparisons test was used in **c**. Data are expressed as mean ± SEM. * $P < 0.05$, ** $P < 0.01$.

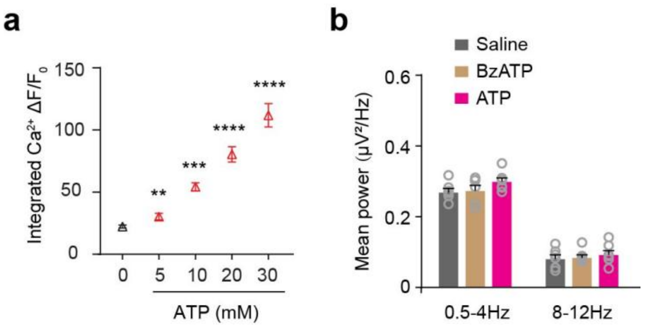

Supplementary Fig. 5| (**a**) Peripheral ATP application enhanced Ca^{2+} activity in L4 DRG neurons in a dose-dependent manner. n = 114 neurons from 3 mice per group. (**b**) ECoG delta and alpha band power in naïve mice with saline, BzATP or ATP treatment for 3 days. n = 6 mice per group. One way ANOVA followed by Dunnett's multiple comparisons test was used in **a**, **b**. Data are expressed as mean ± SEM. * $P < 0.05$, ** $P < 0.01$, *** $P < 0.001$, **** $P < 0.0001$.

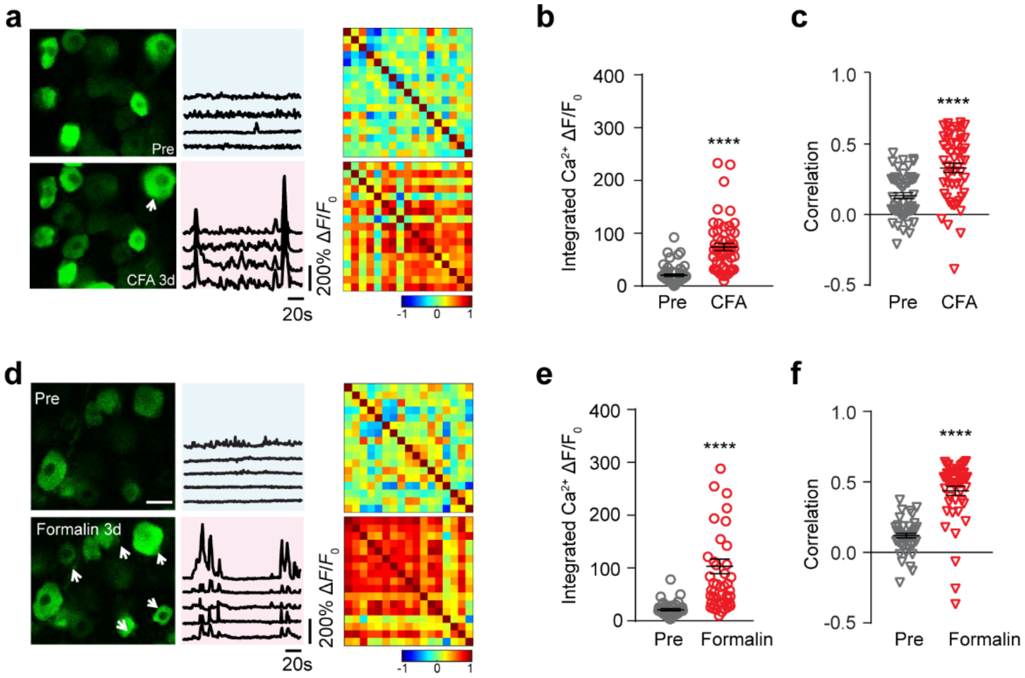

Supplementary Fig. 6| (a-f) DRG activity and synchrony in CFA- and formalin-induced pain. (**a**) Representative images, fluorescence traces and correlation matrices of DRG neurons before and 3 days after plantar CFA injection. (**b-c)** Integrated Ca^{2+} activity (**b**) and correlation coefficient (**c**) of DRG neurons before and 3 days after plantar CFA injections. n = 52 neurons from 3 mice. (**d**) Representative images, fluorescence traces and correlation matrices of DRG neurons before and 3 days after plantar formalin injection. (**e-f)** Integrated Ca^{2+} activity (**e**) and correlation coefficient (**f**) of DRG neurons before and 3 days after plantar formalin injections. n = 46 neurons from 2 mice. Two-tail paired t test was used in **b-c**, **e-f**; Data are expressed as mean ± SEM. **** $P < 0.0001$.

Supplementary tables

Supplementary Table 1. Results of statistical tests, Related to Figures 1-6

Figure	Statistical test	Test statistics	*P* value	n value
1d	One way ANOVA followed by Dunnett's multiple comparisons test	4-8 Hz, F (3, 20) = 5.785	4-8 Hz: pre vs. 3h, *P* = 0.8242; pre vs. 1d, *P* = 0.9175; pre vs. 3d, *P* = 0.0034	*n* = 6 mice per group
1e	Linear regression	R^2 = 0.3612; Y = -65.58*X + 162.6	*P* = 0.0003	*n* = 8 mice per group
1f	NA	NA	NA	*n* = 5 mice per group
1g	One way ANOVA followed by Dunnett's multiple comparisons test	F (3, 724) = 4.877	pre vs. 3h, *P* > 0.999; pre vs. 1d, *P* = 0.3119; pre vs. 3d, *P* = 0.0033	*n* = 5 mice per group; n = 181, 194, 157, 196 neurons, respectively.
1h	NA	NA	NA	*n* = 5 mice per group
1i	One way ANOVA followed by Dunnett's multiple comparisons test	F (3, 724) = 7.313	Pre vs. 3h, *P* =0.9498; Pre vs. 1d, *P* = 0.6859; Pre vs. 3d, *P* = 0.0006	*n* = 5 mice per group; n = 181, 194, 157, 196 neurons, respectively.
1j	Linear regression	R^2 = 0.3223; Y = 177.9*X + 78.64	*P* = 0.0001	*n* = 10 mice per group
2b	NA	NA	NA	*n* = 4 mice per group
2c	One way ANOVA followed by Dunnett's multiple comparisons test	F (3, 270) = 23.97	3h vs. pre, *P* = 0.0396; 1d vs. 3h, *P* = 0.0392; 3d vs. 1d, *P* = 0.0006	*n* = 4 mice per group; n = 117 neurons per group.
2d	NA	NA	NA	*n* = 4 mice per group
2e	One way ANOVA followed by Dunnett's multiple comparisons test	F (3, 504) = 68.17	3h vs. pre, *P* < 0.000000000000001; 1d vs. 3h, *P* = 0.0000267; 3d vs. 1d, *P* = 0.0003	*n* = 4 mice per group; n = 117 neurons per group.
2g	Two-tail paired t test	t_{146} = 14.09	*P* < 0.000000000000001	*n* = 147 neurons from 4 mice
2h	Two-tail paired t test	t_{146} = 15.08	*P* < 0.000000000000001	*n* = 147 neurons from 4 mice
2j	Two-tail paired t test	t_{171} = 4.59	*P* = 0.00000835	*n* = 172 neurons from 5 mice

2k	Two-tail paired t test	$t_{171} = 6.305$	*P* = 0.00000000238	*n* = 172 neurons from 5 mice
2l	Two-tail unpaired *t*-test	$t_{10} = 3.181$	4-8 Hz, *P* = 0.0098	*n* = 6 mice per group
3a	NA	NA	NA	*n* = 3 mice
3b	Two-tail paired t test	t_{38}=4.794	*P* = 0.0000252	*n* = 39 neurons from 3 mice
3c	Two-tail paired t test	t_{38}=6.251	*P* = 0.000000258	*n* = 39 neurons from 3 mice
3d	NA	NA	NA	*n* = 3 mice
3e	Two-tail paired t test	t_{55}=1.349	*P* = 0.1829	*n* = 56 neurons from 3 mice
3f	Two-tail paired t test	t_{55}=0.6437	*P* = 0.5224	*n* = 56 neurons from 3 mice
4b	Two way ANOVA followed by Sidak's multiple comparisons test	$F_{surgery}(1,6) = 287.1$<br><br>$F_{time}(3, 24) = 186.8$	sham vs. SNI<br>0.5h, 3h:<br>*P* < 0.000000000000001;<br>8h: P = 0.000000297;<br>24h: *P* = 0.00510<br>Within SNI<br>0.5h vs. 8h:<br>*P* = 0.000000000000009;<br>0.5h vs. 24h:<br>*P* < 0.000000000000001;<br>3h vs. 8h:<br>*P* = 0.000000000000001;<br>3h vs. 24h,<br>*P* < 0.000000000000001;	*n* = 4 mice per group
4c	One way ANOVA followed by Dunnett's multiple comparisons test	F (3, 32) = 11.861	sham vs. 3h, *P* =0.0134<br>sham vs. 8h, *P* =0.000271<br>sham vs. 1d, *P* = 0.0000355	*n* = 9 mice per group
4e	Two-tail paired t test	$t_{172} = 8.062$;<br>$t_{118} = 3.995$;<br>$t_{131} = 3.182$;<br>$t_{128} = 4.873$	pre vs. ATP, *P* *P* < 0.000000000000001;;<br>pre vs. A-317491, *P* = 0.0001; pre vs. Apyrase, *P* = 0.0018; pre vs. nerve isolation, *P* < 0.000000000000001;	*n* = 4 mice per group; n = 173, 173, 119, 132, 129 neurons, respectively.
4f	Two-tail paired t test	$t_{172} = 7.422$;<br>$t_{118} = 5.256$;<br>$t_{131} = 2.814$;<br>$t_{128} = 5.559$	pre vs. ATP, pre vs. A317491, *P* < 0.000000000000001;;<br>pre vs. apyrase, *P* = 0.0056; pre vs. nerve isolation, *P* < 0.000000000000001;	*n* = 4 mice per group; n = 173, 173, 119, 132, 129 neurons, respectively.
4g	Two-tail paired t test	$t_7 = 6.068$;<br>$t_7 = 5.112$;<br><br>$t_7 = 3.003$;	pre vs. ATP, *P* = 0.0005;<br>pre vs. A-317491,<br>*P* = 0.0014;<br>pre vs. Apyrase,	*n* = 8 mice in SNI,<br>SNI+ATP,<br>SNI+A-

		$t_6 = 5.128$	*P* = 0.0199; pre vs. nerve isolation, *P* = 0.0022	317491 and apyrase groups, n = 7 mice in nerve isolation group
5b	One way ANOVA followed by Sidak's multiple comparisons test	F (2, 476) = 31.3	SNI vs. SNI+ATP, *P* = 0.000000232; SNI vs. SNI+A317491, *P* = 0.0052	*n* = 4 mice per group; n = 196, 158 and 125 neurons, respectively.
5c	One way ANOVA followed by Sidak's multiple comparisons test	F (2, 476) = 147.6	SNI vs. SNI+ATP, *P* < 0.000000000000001; SNI vs. SNI+A317491, *P* = 0.026	*n* = 4 mice per group; n = 196, 158 and 125 neurons, respectively.
5d	One way ANOVA followed by Sidak's multiple comparisons test	4-8 Hz, F (2, 15) = 19.59;	4-8 Hz: SNI vs. SNI+ATP, *P* = 0.005; SNI vs. SNI+A317491, *P* = 0.039	*n* = 6 mice per group
5f	NA	NA	NA	*n* = 4 mice per group
5g	Two way ANOVA followed by Dunnett's multiple comparisons test	F (3,36) = 7.717	3d: sham vs. SNI, *P* = 0.0294; SNI vs. SNI+ ATP, *P* = 0.0082; SNI vs. SNI+A317491, *P* = 0.0253;	*n* = 4 mice per group
5h	Two way ANOVA followed by Dunnett's multiple comparisons test	F (3,36) = 4.05	3d: sham vs. SNI, P = 0.0261; SNI vs. SNI+ ATP, *P* = 0.9987; SNI vs. SNI+A317491, *P* = 0.4998	*n* = 4 mice per group
5i	One way ANOVA followed by Dunnett's multiple comparisons test	F (2, 21) = 18.91	SNI vs. SNI+ATP, *P* = 0.0052; SNI vs. SNI+A-317491, *P* = 0.0226	*n* = 7 mice per group
6c	One way ANOVA followed by Dunnett's multiple comparisons test	F (2, 339) = 39.81	saline vs. BzATP, *P* = 0.00000000000859; saline vs. ATP, *P* = 0.000000000000024; BzATP vs. ATP, *P* = 0.7597	*n* = 3 mice per group; *n* = 114 neurons per group.
6e	One way ANOVA followed by Sidak's multiple comparisons test	F (2, 339) = 70.1	pre vs. BzATP, *P* = 0.7502; pre vs. ATP(10mM), BzATP vs. ATP(10mM), *P* < 0.000000000000001;	*n* = 3 mice per group; *n* = 114 neurons per group.
6g	One way ANOVA followed by Sidak's multiple comparisons test	F (2, 512) = 6.586	saline vs. BzATP, *P* = 0.8634; saline vs. ATP, *P* = 0.0028	*n* = 4 mice per group; n = 143, 178 and 194 neurons, respectively.
6h	One way ANOVA followed by Sidak's multiple comparisons test	F (2, 512) = 10.54	saline vs. BzATP, *P* = 0.8605; saline vs. ATP, *P* = 0.0001	*n* = 4 mice per group; n = 143, 178 and 194 neurons, respectively.

6j	One way ANOVA followed by Dunnett's multiple comparisons test	4-8 Hz, $F(2, 15) = 11.21$	4-8 Hz: saline vs. BzATP, $P = 0.9491$; saline vs. ATP, $P = 0.0024$	$n = 4$ mice per group
6l	Two way ANOVA followed by Dunnett's multiple comparisons test	$F_{treatment}(2, 27) = 3.644$	3d: saline vs. BzATP, $P = 0.1524$; saline vs. ATP, $P = 0.0025$	$n = 4$ mice per group
6m	Two way ANOVA followed by Dunnett's multiple comparisons test	$F_{treatment}(2, 27) = 5.28$	3d: saline vs. BzATP, $P = 0.3373$; saline vs. ATP, $P = 0.4331$	$n = 4$ mice per group
6n	One way ANOVA followed by Dunnett's multiple comparisons test	$F(2, 21) = 7.827$	saline vs. BzATP, $P = 0.7543$; saline vs. ATP, $P = 0.0025$;	$n = 8$ mice per group
6o	One way ANOVA followed by Dunnett's multiple comparisons test	$F(2, 18) = 6.197$	saline vs. BzATP, $P = 0.9422$; saline vs. ATP, $P = 0.0097$	$n = 7$ mice per group
