# Supplementary material for: Synchronized activity of sensory neurons initiates cortical synchrony in a model of neuropathic pain
Source: Nat Commun. 2023 Feb 8;14:689. doi: 10.1038/s41467-023-36093-z (PMC9908980; doi:10.1038/s41467-023-36093-z)
Supplement: Supplementary file 2 — Description of Additional Supplementary Files [file 41467_2023_36093_MOESM2_ESM.pdf]

Description of Additional Supplementary Files

Supplementary Movie 1. Calcium activity of DRG sensory neurons before and after SNI.

Supplementary Movie 2. Calcium activity of DRG sensory neurons under resting awake state.

Supplementary Movie 3. BzATP activates DRG sensory neurons in asynchrony.

Supplementary Movie 4. ATP activates DRG sensory neurons in synchrony.

Supplementary Software 1. Matlab code for ECoG analysis, Demo data and step-by-step protocol are provided in the .zip file.
