# Supplementary material for: Synchronized activity of sensory neurons initiates cortical synchrony in a model of neuropathic pain
Source: Nat Commun. 2023 Feb 8;14:689. doi: 10.1038/s41467-023-36093-z (PMC9908980; doi:10.1038/s41467-023-36093-z)

1. System requirements

Matlab version: R2020b, Toolbox needed:

operating system: windows 10

No non-standard hardware required

1. Installation guides

Typical recommended installation

Unzip the file and put the folder under C:\Lab\MATLAB\EEG\code

- 1. Demo for run the data

1. In matlab, add the above folder to the path


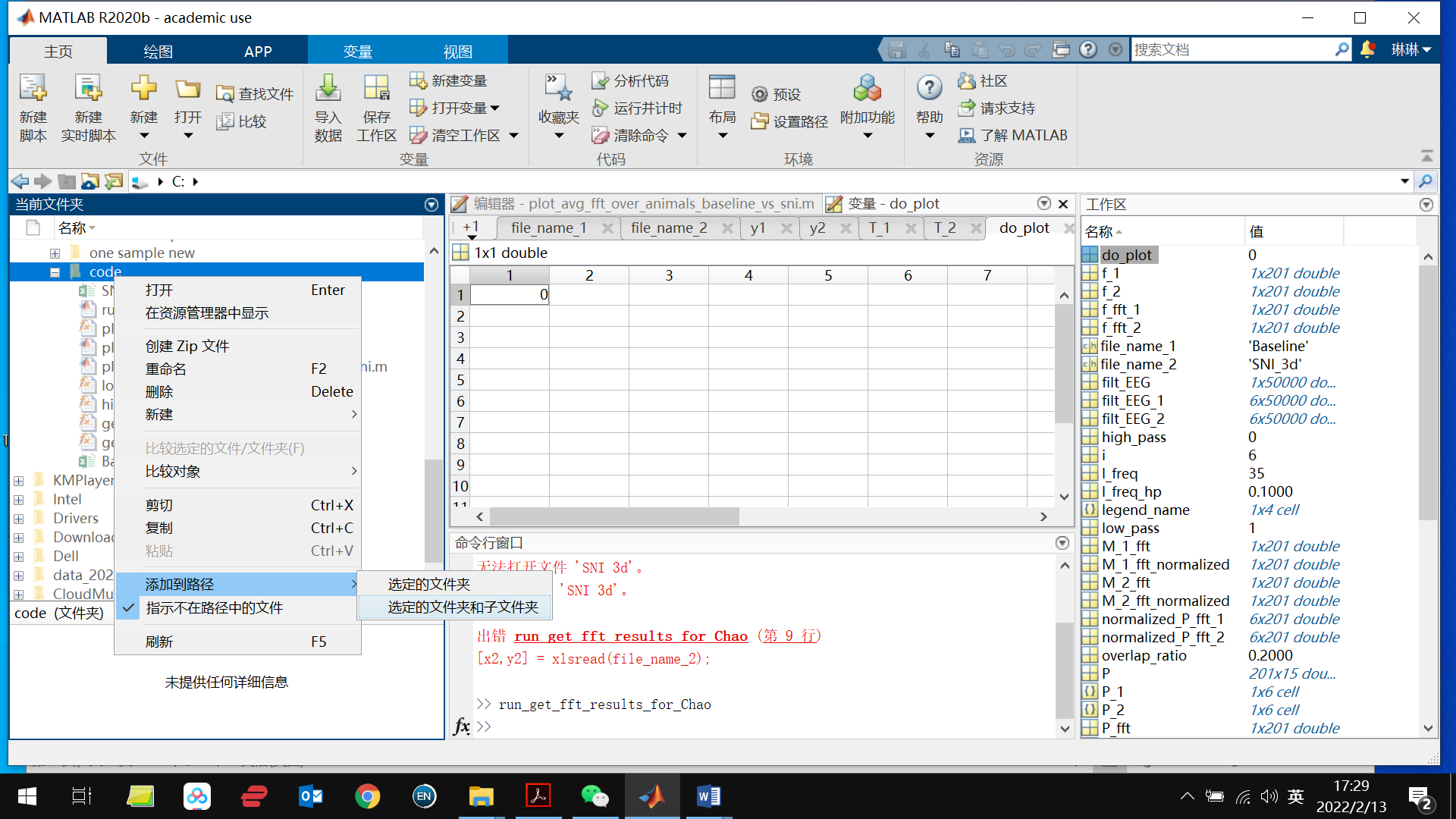


1. Click the function run_get_fft_results_for_synchrony.m and run it, this will get the EEG power spectrum for Saline and ATP group (the two excel sheets are included in the folder, each includes EEG original signal recorded from 6 animals).


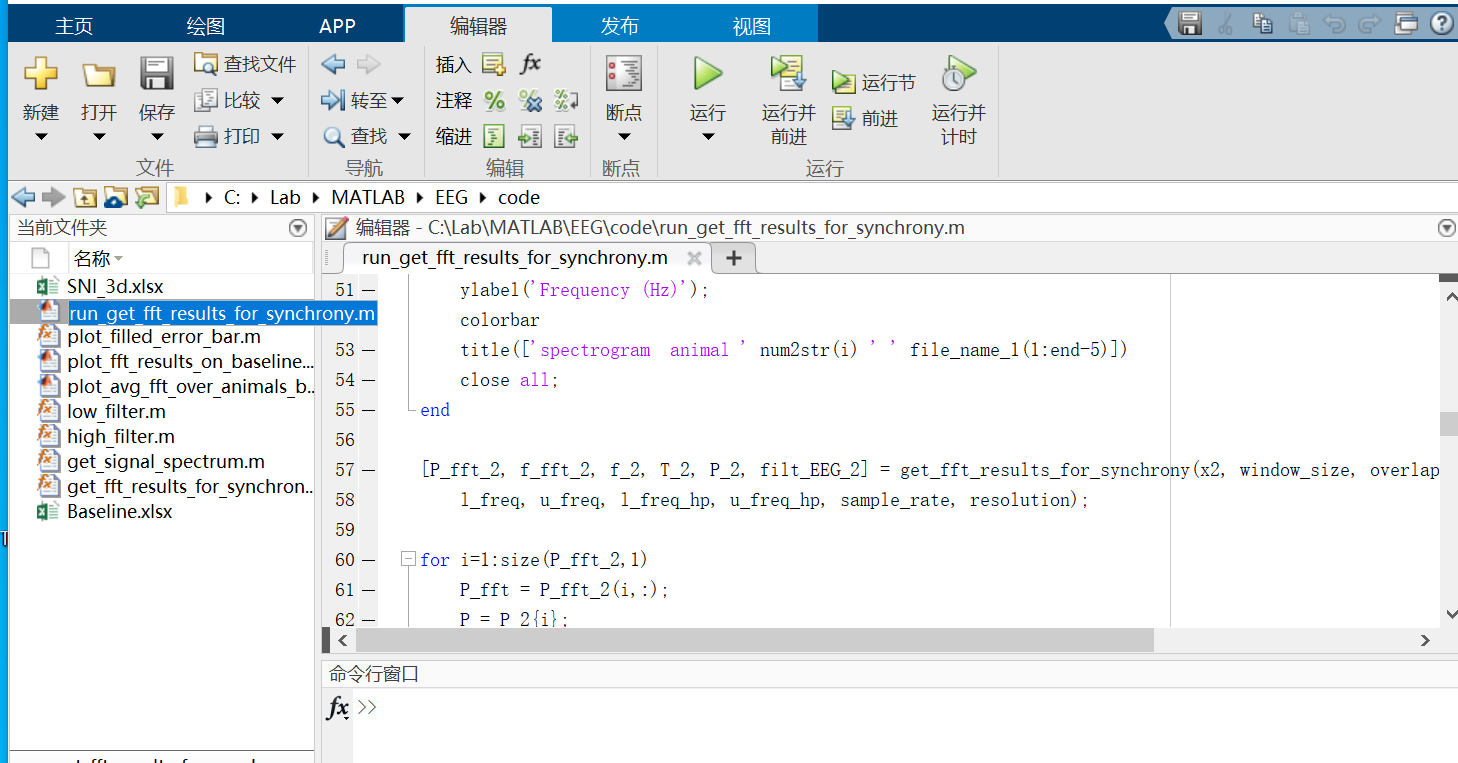


1. This will get the EEG spectrum data


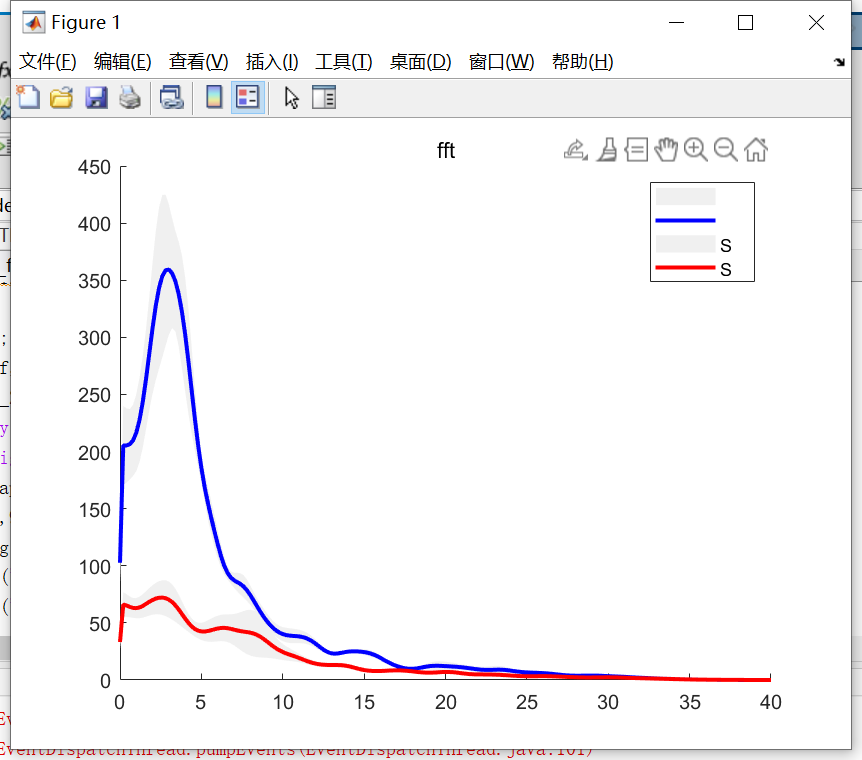

Supplement: Supplementary file 7 — Supplementary Software 1 [file 41467_2023_36093_MOESM7_ESM.zip › code/Readme.docx]
