# Supplementary material for: Synchronized activity of sensory neurons initiates cortical synchrony in a model of neuropathic pain
Source: Nat Commun. 2023 Feb 8;14:689. doi: 10.1038/s41467-023-36093-z (PMC9908980; doi:10.1038/s41467-023-36093-z)
Supplement: Supplementary file 8 — Reporting Summary [file 41467_2023_36093_MOESM8_ESM.pdf]

nature portfolio

Corresponding author(s): Wen-biao Gan

Last updated by author(s): Dec 21, 2022

Reporting Summary

Nature Portfolio wishes to improve the reproducibility of the work that we publish. This form provides structure for consistency and transparency in reporting. For further information on Nature Portfolio policies, see our Editorial Policies and the Editorial Policy Checklist.

Statistics

For all statistical analyses, confirm that the following items are present in the figure legend, table legend, main text, or Methods section.

n/a	Confirmed	
☐	☒	The exact sample size (n) for each experimental group/condition, given as a discrete number and unit of measurement
☐	☒	A statement on whether measurements were taken from distinct samples or whether the same sample was measured repeatedly
☐	☒	The statistical test(s) used AND whether they are one- or two-sided<br>*Only common tests should be described solely by name; describe more complex techniques in the Methods section.*
☐	☒	A description of all covariates tested
☐	☒	A description of any assumptions or corrections, such as tests of normality and adjustment for multiple comparisons
☐	☒	A full description of the statistical parameters including central tendency (e.g. means) or other basic estimates (e.g. regression coefficient) AND variation (e.g. standard deviation) or associated estimates of uncertainty (e.g. confidence intervals)
☐	☒	For null hypothesis testing, the test statistic (e.g. F, t, r) with confidence intervals, effect sizes, degrees of freedom and P value noted<br>*Give P values as exact values whenever suitable.*
☒	☐	For Bayesian analysis, information on the choice of priors and Markov chain Monte Carlo settings
☒	☐	For hierarchical and complex designs, identification of the appropriate level for tests and full reporting of outcomes
☐	☒	Estimates of effect sizes (e.g. Cohen's d, Pearson's r), indicating how they were calculated

Our web collection on statistics for biologists contains articles on many of the points above.

Software and code

Policy information about availability of computer code

Data collection	ANY-Maze 6.0: open field and CPP; Bruker PrairieView 4.0: Imaging data acquisition
Data analysis	Image J Fiji 1.49U: imaging analysis; ANY-Maze 6.0: open field and CPP analysis; GraphPad Prism 7: statistical tests

For manuscripts utilizing custom algorithms or software that are central to the research but not yet described in published literature, software must be made available to editors and reviewers. We strongly encourage code deposition in a community repository (e.g. GitHub). See the Nature Portfolio guidelines for submitting code & software for further information.

Data

Policy information about availability of data

All manuscripts must include a data availability statement. This statement should provide the following information, where applicable:

- Accession codes, unique identifiers, or web links for publicly available datasets
- A description of any restrictions on data availability
- For clinical datasets or third party data, please ensure that the statement adheres to our policy

The data that support the findings of this study are all provided in the Supplementary Information and Source Data File.

Field-specific reporting

Life sciences study design

All studies must disclose on these points even when the disclosure is negative.

Sample size	No statistical methods were used to pre-determine sample sizes. Our sample sizes are similar to previously reported publications. For behavioral tests, 8~12 mice per group are used; for molecular tests, 6-10 mice per group are used; for Imaging experiments, 3-5 mice per group are used.
Data exclusions	No samples or animals that were successfully imaged or measured were excluded from the analysis.
Replication	We use biological repeats, hence each experiment was performed in different mice or samples from different mice, and analysis was made pulling all data together. The exclusion would be made if a data point ranges out of 3 times group SD. In our current study, no data were excluded from the analysis.
Randomization	All animals were randomly assigned to experimental groups. In CPP tests, in which the side and the order of injections were further counterbalanced across animals.
Blinding	Experimenters were blinded to group allocations in all experiments.

Reporting for specific materials, systems and methods

We require information from authors about some types of materials, experimental systems and methods used in many studies. Here, indicate whether each material, system or method listed is relevant to your study. If you are not sure if a list item applies to your research, read the appropriate section before selecting a response.

Materials & experimental systems

n/a	Involved in the study	
☐	☒	Antibodies
☒	☐	Eukaryotic cell lines
☒	☐	Palaeontology and archaeology
☐	☒	Animals and other organisms
☒	☐	Human research participants
☒	☐	Clinical data
☒	☐	Dual use research of concern

Methods

n/a	Involved in the study	
☒	☐	ChIP-seq
☒	☐	Flow cytometry
☒	☐	MRI-based neuroimaging

Antibodies

Antibodies used	Anti-P2x3r, Sigma, ab5895, 1:500; Anti-β-actin, Cell Signalling Technology, #3700, 1:2000; Anti-P2x3r, Santa Cruz, sc-390572, 1:500; anti-mouse-IgG-HRP, Santa Cruz, 7076S, 1:1000; mouse anti-rabbit IgG-HRP, Santa Cruz, sc-2357, 1:1000;
Validation	Antibodies used in this study were all validated in mice samples by the manufacturer on the website. We tested our primary antibodies in this study in the DRG nerve fiber samples. Obvious bands for P2X3r and β-actin were observed at the correct location and backgrounds were clear.

Animals and other organisms

Policy information about studies involving animals; ARRIVE guidelines recommended for reporting animal research

Laboratory animals	Mouse, Thy1-YFP-H line, male and female, two- to 3-month old; Mouse, Thy1-GCaMP6 slow founder line 3, male and female, two- to 3-month old; Mouse, wildtype C57BL6J (Jackson Laboratory; stock no. 000664), male and female, two- to 3-month old
Wild animals	This study did not involve wild animals.
Field-collected samples	This study did not involve samples collected from the field.
Ethics oversight	Study protocols have been approved by the Institutional Animal Care and Use Committee (IACUC) of both New York University and Columbia University as consistent with National Institutes of Health (NIH) Guidelines for the Care and Use of Laboratory Animals.

Note that full information on the approval of the study protocol must also be provided in the manuscript.
